# Dynamic distribution decomposition for single-cell snapshot time series identifies subpopulations and trajectories during iPSC reprogramming

**DOI:** 10.1371/journal.pcbi.1007491

**Published:** 2020-01-10

**Authors:** Jake P. Taylor-King, Asbjørn N. Riseth, Will Macnair, Manfred Claassen

**Affiliations:** 1 Institute of Molecular Systems Biology, Department of Biology, ETHZ, Zurich, Switzerland; 2 Juvenescence AI, Viking House, Nelson Street, Douglas, Isle of Man, United Kingdom; 3 Mathematical Institute, University of Oxford, Oxford, United Kingdom; 4 Institute of Molecular Life Sciences, University of Zurich, Zurich, Switzerland; University of California Irvine, UNITED STATES

## Abstract

Recent high-dimensional single-cell technologies such as mass cytometry are enabling time series experiments to monitor the temporal evolution of cell state distributions and to identify dynamically important cell states, such as fate decision states in differentiation. However, these technologies are destructive, and require analysis approaches that temporally map between cell state distributions across time points. Current approaches to approximate the single-cell time series as a dynamical system suffer from too restrictive assumptions about the type of kinetics, or link together pairs of sequential measurements in a discontinuous fashion. We propose Dynamic Distribution Decomposition (DDD), an operator approximation approach to infer a continuous distribution map between time points. On the basis of single-cell snapshot time series data, DDD approximates the continuous time Perron-Frobenius operator by means of a finite set of basis functions. This procedure can be interpreted as a continuous time Markov chain over a continuum of states. By only assuming a memoryless Markov (autonomous) process, the types of dynamics represented are more general than those represented by other common models, e.g., chemical reaction networks, stochastic differential equations. Furthermore, we can *a posteriori* check whether the autonomy assumptions are valid by calculation of prediction error—which we show gives a measure of autonomy within the studied system. The continuity and autonomy assumptions ensure that the same dynamical system maps between all time points, not arbitrarily changing at each time point. We demonstrate the ability of DDD to reconstruct dynamically important cell states and their transitions both on synthetic data, as well as on mass cytometry time series of iPSC reprogramming of a fibroblast system. We use DDD to find previously identified subpopulations of cells and to visualise differentiation trajectories. Dynamic Distribution Decomposition allows interpretation of high-dimensional snapshot time series data as a low-dimensional Markov process, thereby enabling an interpretable dynamics analysis for a variety of biological processes by means of identifying their dynamically important cell states.

## Author summary

High-dimensional single-cell snapshot measurements are now increasingly utilized to study dynamic processes. Such measurements enable us to evaluate cell population distributions and their evolution over time. However, it is not trivial to map these distribution across time and to identify dynamically important cell states, i.e. bottleneck regions of state space exhibiting a high degree of change. We present Dynamic Distribution Decomposition (DDD) achieving this task by encoding single-cell measurements as linear combination of basis function distributions and evolving these as a linear system. We demonstrate reconstruction of dynamically important states for synthetic data of a bifurcated diffusion process and mass cytometry data for iPSC reprogramming.

## Introduction

Data-driven reconstruction of dynamic processes constitutes a central aim of systems biology. High-dimensional single-cell molecularly resolved time series data is becoming a key data source for this task [1, 2]. However, these technologies are destructive, and consequently result in *snapshot time series* data originating from batches of cells collected at time points of interest. A longstanding and still challenging problem is to reconstruct dynamic biological processes from this data, to the end of identifying dynamically important states, i.e. regions of state space that cells preferentially pass through, these include transitionary states (bottlenecks) when differentiation decisions are made, and terminal states. With *snapshot time series*, it is challenging to identify these states as we cannot track the state of an individual cell from one time point to a new state at a later time point, one has to temporally map between distributions over the state space.

Chemical reaction networks (CRNs) are a popular class of parametric models assuming that the temporal state evolution is well described by chemical kinetics. Ordinary differential equations (ODEs) are used to describe smooth deterministic dynamics, and stochastic differential equations (SDEs) for dynamics in the low copy number/concentration regimes affected by stochastic fluctuations. Chemical reaction network models require explicit definition of the model structure, i.e. set of reactions or interactions among the system components. This task is manageable for small, well defined systems, such as small signalling systems [3]. However, by means of high-dimensional measurements, we typically observe larger systems comprising at least dozens of components with largely *a priori* undefined interactions. This situation results in a combinatorial explosion of model variants that cannot be exhaustively evaluated [4, 5]. Alternative approaches are agnostic with regards to parametric form and model structure and use a probabilistically-motivated rule to map between distributions, e.g., one optimal transport method maps neighbours at one time point to the nearest neighbour at the next time point [6]. However, such generic approaches are rather extreme in their agnosticism and abandon reasonable assumptions on the dynamics of cellular systems, e.g., that cells can be modelled as an *autonomous* dynamical systems in continuous time, such as a Markov chain, where the cell’s current state infers its likely future state independent of the current time within the experiment. Such autonomy assumptions are also commonly used in many mechanistic CRN, SDE and ODE biochemical models [7, 8].

Operator approximation methods constitute an alternative class of models that are agnostic to model structure and yet allow for encoding of general system properties such as autonomy, conservation of mass, and boundary conditions. These methods approximate both the Perron–Frobenius operator [9] and the Koopman operator [10]. These operators describe the evolution of distributions and other functions of a dynamical system’s state. The early theory on these operators was developed to describe systems in classical, statistical, and quantum mechanics [11–15], and in probability theory [16, 17]. The operators fully describe a nonlinear dynamical system as a linear system in higher, possibly infinite, dimensions. Hence, techniques from linear analysis can be utilised to gain insight from the systems; in particular, calculation of eigenvalues and eigenfunctions allow for timescale separation. Eigenfunctions of linear operators show the fundamental building blocks of possible behaviours available to a dynamical system, e.g., exponential growth/decay, oscillations, a steady state. Data-driven approximations to the operators have been investigated in past years, originating in the computational fluid dynamics community [18–22]. Their focus is to approximate finite-dimensional projections of the Koopman operator with a family of algorithms known as Dynamic Mode Decomposition (DMD). The algorithm has further been applied to other areas such as neuroscience, infectious disease epidemiology, and control theory [23–25] and parameter estimation [26, 27]. When carrying out approximations of these operators, eigenvalues can be ordered in terms of magnitude to extract slow behaviour (approximated well) to fast behaviour (representative of noise) [27]. Dynamic Mode Decomposition assumes that the data is recorded at equally spaced time points, whilst our work extends the technique to support data recorded at arbitrary time points.

We adapt Dynamic Mode Decomposition to identify dynamically important states from single-cell snapshot time series. Our method is based on representing the distribution at each time point via basis functions and calculating an approximation to the Perron–Frobenius operator by minimising an error term—akin to least squares when fitting ODEs. The error terms then indicate how well the data fits into the model assumptions *a posteriori*: primarily that the data is generated by an autonomous dynamical system. Because the Perron–Frobenius operator describes the evolution of distributions, we name our approach Dynamic Distribution Decomposition (DDD) in keeping with the DMD naming convention. DDD leads to the calculation of a Markov transition rate matrix but over a continuum of states—as opposed to discrete states. As previously mentioned, one can then use standard methods of analysis for linear operators based on eigen-decompositions. As Markov processes can be represented as directed weighted graphs, our graph can be evaluated in two dimensions and then the high-dimensional operator and its corresponding eigenfunctions have a natural low dimensional representation. Our approach also allows for visualisation of inferred state trajectories as a branching structure when cell fates are stochastic, and approximation of fitting error when matching model prediction to sample data. By using a Markov rate approach over distributions, we overcome the difficulties listed above. In particular: our approach generalises the previously listed mechanistic models (CRNs, SDEs, ODEs) and works similarly to optimal transport methods—however, allowing for fitting error and not achieving having perfect fits by interpolating between time points. We demonstrate DDD on a synthetic stochastic dynamical system representing cells making a cell differentiation decision as well as for a mass cytometry time series taken from an iPSC reprogramming of a fibroblast cell line taken from Zunder *et. al*. [28] to re-identify subpopulations of cells as first elucidated in the original manuscript.

## Results

### Inference of state distribution dynamics by approximation of the Perron–Frobenius operator

**Fig 1:**
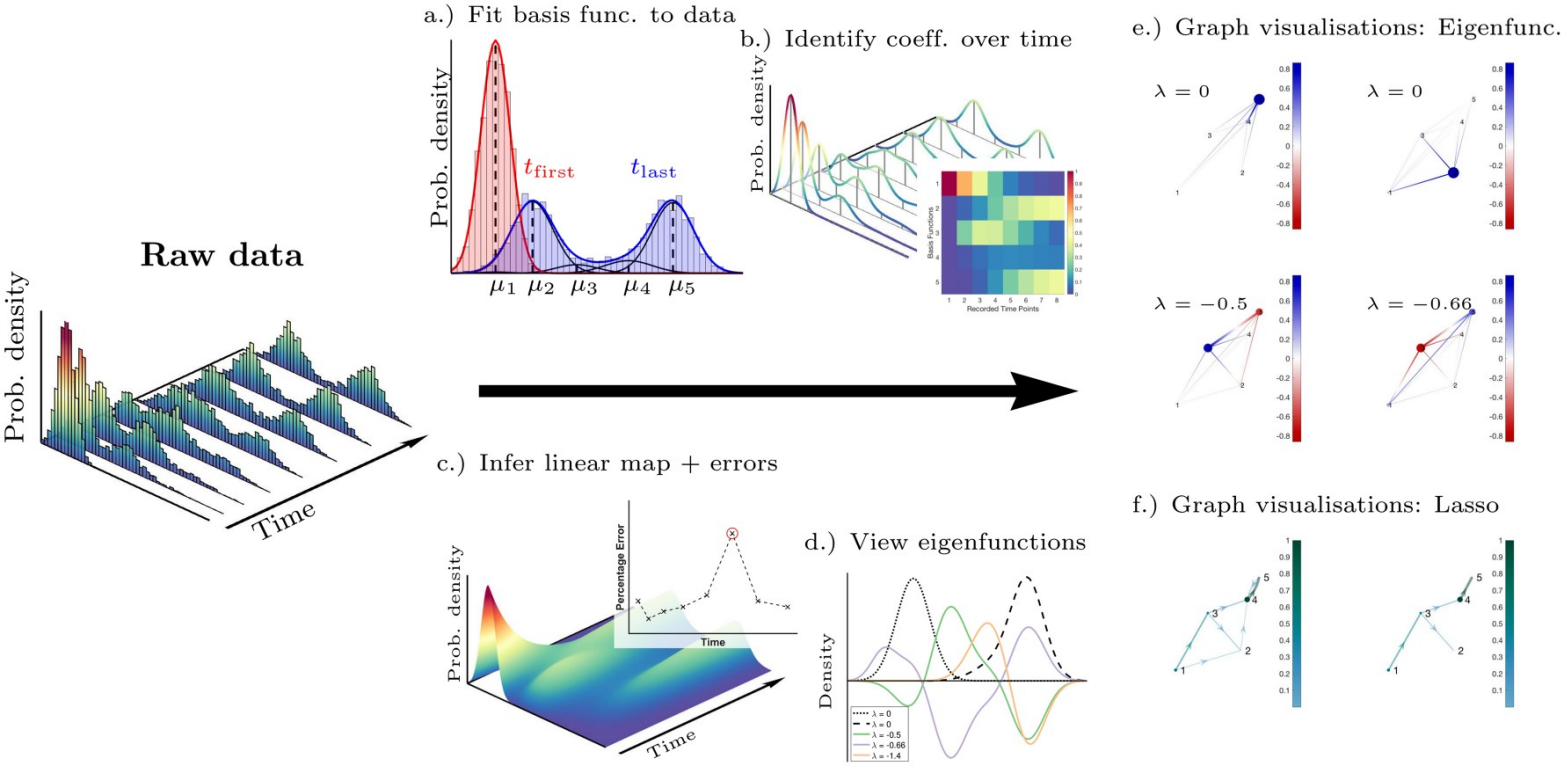
Illustration of Dynamic Distribution Decomposition workflow. (a.) Gaussian mixture models as basis functions: single-cell profiles from each time point are fitted to a basis of Gaussian mixture model, two distributions shown in red and blue; (b.) identification of the coefficients ***c***(*t*) at each time point, *t* = *t*_1_, …, *t*_*R*_; (c.) the Perron–Frobenius matrix *P* enables us us to infer the likely state for all time points and generate errors *ε*_1_, …, *ε*_*R*_; (d.) examination of the eigenfunctions in low dimensions; or in high dimensions (e.) using graphical visualisations; and (f.) a Lasso regularisation can reveal sparse structure.

We developed a method herein referred to as Dynamic Distribution Decomposition to analyse snapshot time series, consisting of the following stages (illustrated in Fig 1): (*a*.) data at each recorded time point *t*_1_, …, *t*_*R*_ are fitted to a set of basis functions and (*b*.) encoded into coefficient vectors ***c***_1_, …, ***c***_*R*_; (*c*.) a fitting procedure is carried out to infer the most likely continuous linear map between the coefficients, generating fitting errors *ε*_1_, …, *ε*_*R*_; (*d*.) eigenfunctions are then analysed; and (*e*.) in high dimensions graph based visualisations can be used for eigenfunctions, for full details see Methods section. In the case where probability density functions are used as basis functions, the linear map denoted *P* can be interpreted as a transition rate matrix; the structure of this matrix is often dense but its dominating structure can be elucidated via Lasso regularisation, see Fig 1(f). We applied our method to two systems: first, simulated particles in a potential well; and second, experimental data of iPSC reprogramming of a fibroblast system.

### Particles in potential well with fluctuations

The first numerical example is for illustrative purposes whereby we know the stochastic process generating the sample points. We consider simulated particles in a bistable potential well undergoing fluctuations. After initialisation around point (1, 1)^⊺^/2, particles stochastically switch between one of two paths: *y* = 2*x* or *y* = *x*/2 to finally settle in one of the two final state (2, 4)^⊺^ or (4, 2)^⊺^. We model this process by the two-dimensional SDE $\left\{X_{t}={\left(X_{1t},X_{2t}\right)}^{⊺}\in R_{+}^{2}:t\geq 0\right\}$

$$\begin{array}{c} \text{d}X_{t}=-\nabla V\left(X_{t}\right)\text{d}t+\sqrt{2D}\;\text{d}W_{t}\;, \end{array}$$ (1)

where ***W***_*t*_ is a two-dimensional Wiener process. The potential well is of the form

$$\begin{array}{cc} V(x) & ={\left(\frac{1}{2}{[∥x∥}^{2}-x_{1}x_{2}]-\frac{1}{10}{∥x∥}^{2}\right)}^{2}-\frac{1}{2}e^{-\frac{1}{2}{∥x-{\left(\frac{1}{2},\frac{1}{2}\right)}^{⊺}∥}^{2}}-e^{{-∥x-\left(4,2\right)}^{⊺}{∥}^{2}}-e^{{-∥x-\left(2,4\right)}^{⊺}{∥}^{2}}\;. \end{array}$$ (2)

**Fig 2:**
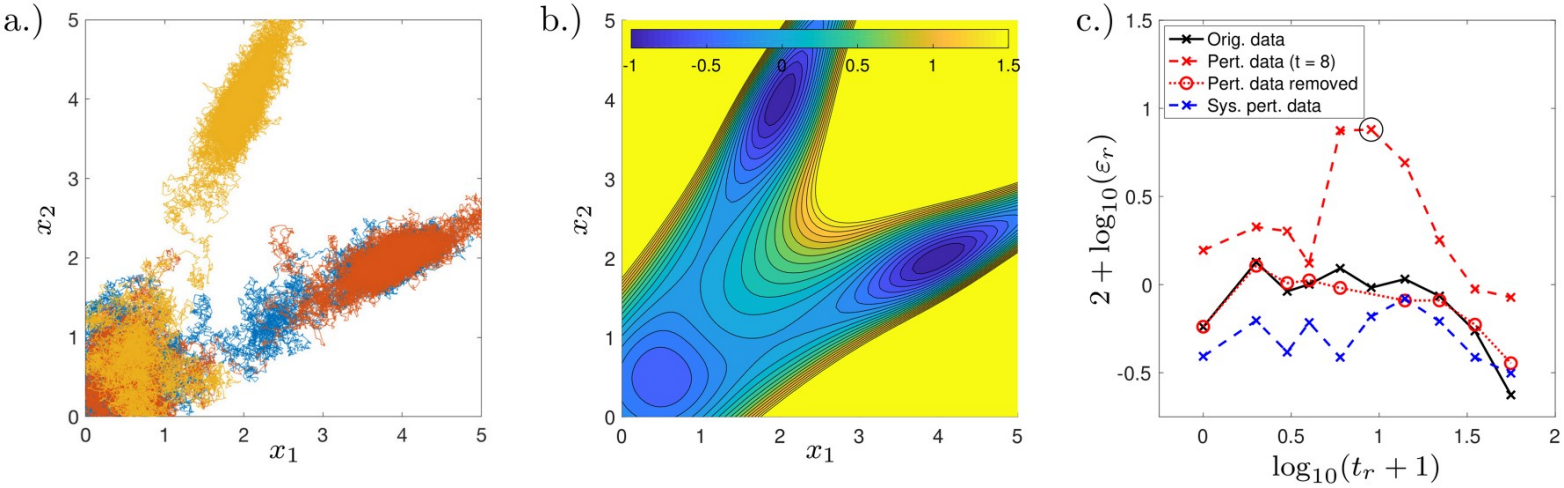
Illustration of stochastic dynamical system described by Eqs (1) and (2) and fitting error of Dynamic Distribution Decomposition process. (a.) Three simulated trajectories are shown from the stochastic dynamical system described by Eqs (1) and (2). (b.) The potential well as described in Eq (2) is shown. (c.) Log-log error plot showing recorded time points against log_10_ of the percentage error (of the DDD fit); the original data set (black solid line) has a mean error of 0.9%; the perturbed data set (dashed red line) has a mean error of 3.1%; the perturbed data set with the erroneous data point removed (dotted red line) has an error of 0.8%; and the data set with systematic error (dashed blue line) has a mean error of 0.5%.

As an initial condition at *t* = *t*_1_, the sample is placed with a multivariate normal distribution with mean ***μ*** = (1, 1)^⊺^/2 and covariance matrix Σ = *I*_2_/2 where *I*_2_ is the identity matrix. The diffusion constant is chosen to be *D* = 1/4. Along the lines *x* = 0 and *y* = 0, the system has reflecting boundaries imposed. For simulations, the Euler–Maruyama (EM) numerical scheme is used with time step *δt* = 2^−9^; for this system the EM scheme is identical to the Milstein scheme and is therefore of order 1. Three sample trajectories are visualised, see Fig 2(a); and the potential well is plotted, see Fig 2(b).

The system is observed at time points *t* = 0, 1, 2, 3, 5, 8, 13, 21, 34, 55 and for each time point 2000 trajectories are initiated at *t* = 0 and simulated until the observation time (replicating the destructive sampling process). Using Gaussian mixture models, we use 3 components for each time point totalling *N* = 30 basis functions. For an illustration showing the entries of the coefficient vectors ${\left\{c_{r}\right\}}_{r=1}^{R}$, see the S2 Appendix.

#### Extrema of eigenfunctions identify steady state and bistable paths

**Fig 3:**
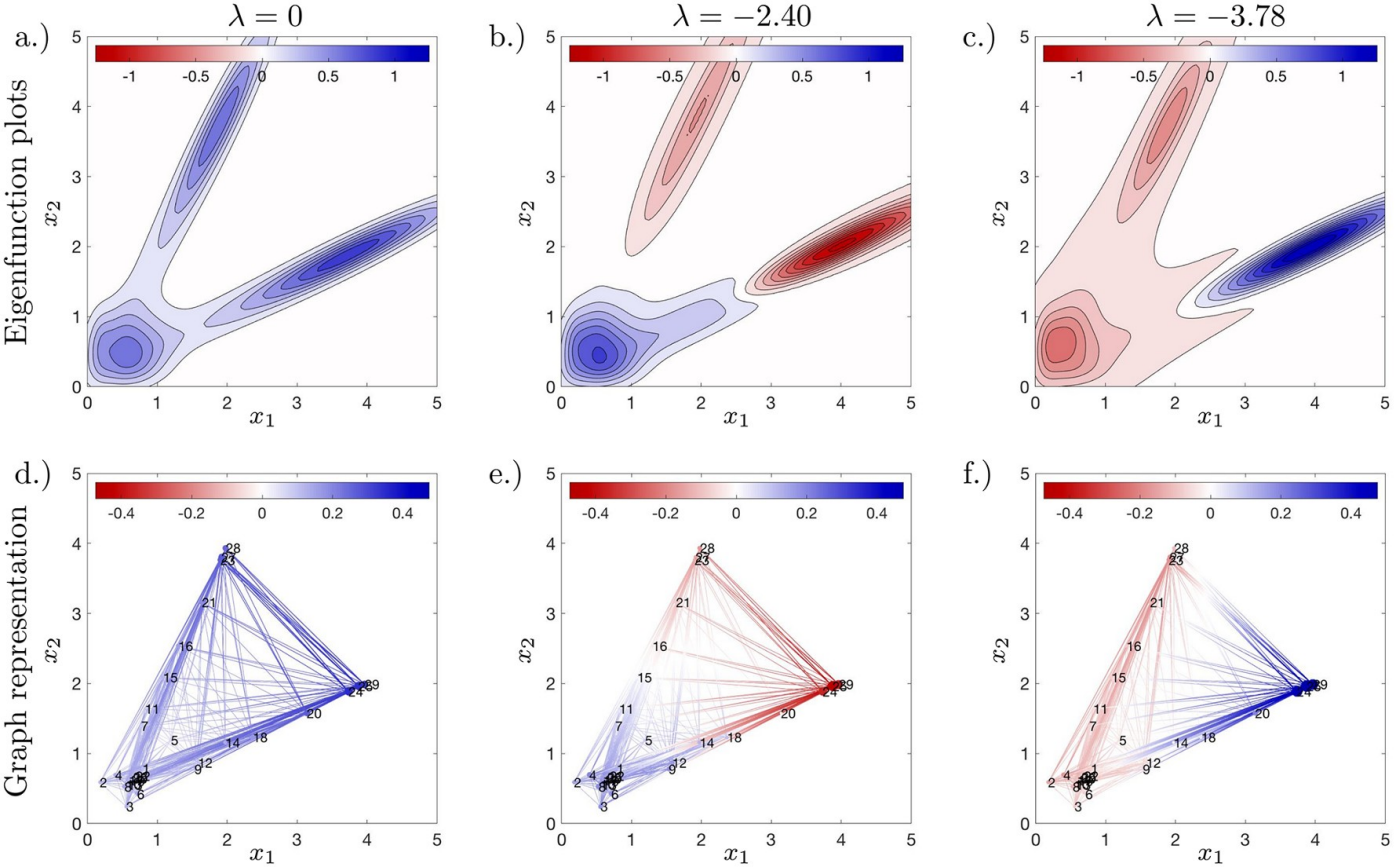
Dynamic distribution decomposition applied to data generated by stochastic dynamical system described by Eqs (1) and (2). Plots (a)–(c) show a plot of the two dimensional eigenfunction, and plots (d)–(f) shows a corresponding graph representation of the eigenfunctions, for full details see Methods section.

The eigenfunctions of the approximated Perron–Frobenius operator allow us to identify the steady state and bistable paths in the above system. In low dimensions we visualise eigenfunctions of *P* as a continuous function, see Fig 3(a), 3(b) and 3(c); or a graph, see Fig 3(d), 3(e) and 3(f) and Methods section. Eigenfunctions corresponding to eigenvalues with large absolute value are approximated with larger error than in cases with small eigenvalues, a point also noted in Ref. [27]; notice in Fig 3 that the eigenfunctions become less (anti-)symmetric along *y* = *x* as the size of |λ| increases. Also, eigenvalues and eigenfunctions are basis function dependent, so changes in basis functions change the eigen-decomposition. However, regardless of changes to the basis functions, the key dynamic states (as visible in the eigenfunctions) remain the same provided the changes to the basis functions are not drastic. Since we use 30 basis functions, hypothetically we can find 30 eigenfunctions. However, we just plot the first three eigenfunctions; these are real with no imaginary component. From these figures, it is clear that three basins around (2, 4)^⊺^ and (4, 2)^⊺^ and at the initial condition (1, 1)^⊺^/2 are dynamically important. Therefore, examination of the first few eigenfunctions allows for detection of dynamically important states.

#### Dynamic distribution decomposition is robust to noisy observations

We evaluated the robustness of our inference procedure to measurement noise. Specifically, three further modified data sets are also considered: (*i*.) considering a single time point perturbed by additive random noise; (*ii*.) removing this perturbed time point and fitting the model; and (*iii*.) randomly perturbing all time points by additive random noise. For all perturbations, sample points are modified by an additive error term drawn from a zero-mean multivariate Gaussian with covariance matrix Σ = *I*_2_/4.

We plot the time points transformed by log_10_(*x* + 1) against the log-percentage error, i.e., 2 + log_10_(*ε*_*r*_) for *r* = 1, …, *R*, see Fig 2(c). We find that all data sets have consistently low error, but with a small increase in error at the beginning of the realisation; this is due to the boundary conditions which were not incorporated into the choice in basis functions, which one would typically do when solving PDEs via a Galerkin approximation. The data set perturbed at time *t* = 8 (dashed red line) leads to increased error immediately before and after this time point (circled in black); after removing this erroneous data (fitting without *t* = 8) one obtains reduced errors comparable to the original data set (dotted red line). When adding systematic error to all time points (dashed blue line), one observes similar (slightly smaller) errors to the original data set; the reason for this is that the Gaussian basis functions now have a covariance matrix with larger entries (whilst the means remain similar) leading to smaller *L*^2^ errors, see Eq (19). The eigenfunction plots are also similar to those generated by the original data set but more spread out (not plotted). In summary: adding noise to a single time point allows for detection of non-autonomous behaviour; adding noise to all time points makes the process appear more random (and hence autonomous); and therefore DDD is robust with regards to noisy observations.

#### Lasso regularisation reveals sparse topology

**Fig 4:**
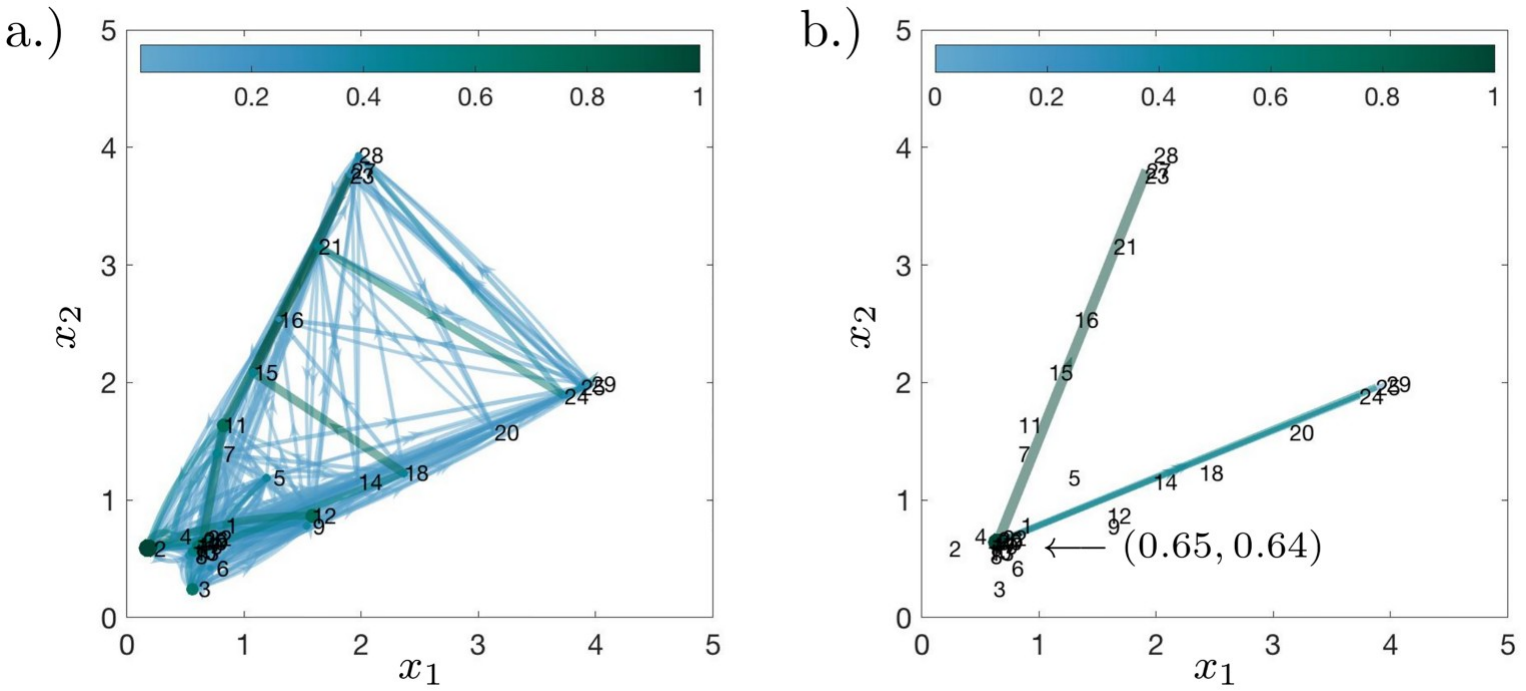
Dynamic distribution decomposition applied to data generated by stochastic dynamical system described by Eqs (1) and (2). The plots show graph representations of *P* (interpreted as a Markov transition rate matrix), colours of edges denote magnitude of rate change from one node to another, and node colours denote rate at which node decays. Colours scaled to unit interval. (a.) Matrix *P* plotted without Lasso regularisation; and (b.) matrix *P* plotted with Lasso regularisation, *β* = 1/[100 × mean(*M*)], see Methods section.

We utilize Lasso regularization to identify key transition states, see Methods section. Specifically, *P* as a transition rate matrix with the nodes located at the mean of the components of the Gaussian mixture model. The resulting network is cluttered and is hard to identify meaningful states or transition, see Fig 4(a). Lasso regularisation encourages sparsity and reveals the simple underlying structure, see Fig 4(b). The skeletal structure shows that around the initial condition the particle becomes strongly committed to one branch over the other—an accurate reflection of the dynamical system.

### Mass cytometry data: iPSC fibroblast reprogramming

We studied the process of iPSC reprogramming using Dynamic Distribution Decomposition. We considered data from a study established by Zunder *et. al*. [28]. Specifically, the reprogramming of a fibroblast cell line differentiating into an induced pluripotent stem cell state was studied using mass cytometry. This *in vitro* experimental system is often perceived to be representative of an autonomous system [8]. However, we can use DDD to suggest when this may or may not be true.

Cells are labelled using mass-tag cell barcoding, stained with antibodies before being measured via CyTOF. We focus our study to the cell line with the largest amount of cell events, i.e. on a Nanog-Neo secondary mouse embroyic fibroblasts (MEF) that expresses neomycin resistance gene from the endogenous Nanog locus. Reprogramming was monitored by Dox induction for 16 days followed by subsequent addition of LIF; the experiment was carried out over 30 days. Experiments were initialised together and cells harvested every 2 days until 24 days with a final measurement taken at the final time point; 18 protein markers were used as proxies to measure pluripotency, differentiation, cell cycle status, and cellular signalling.

We now briefly state our method for choosing basis functions. In the synthetic data example, we used prior information that there were 3 clusters, so used a 3 component Gaussian mixture model for each time (therefore *N* = 30 for 10 time points). For non-synthetic data we do not necessarily have this information, therefore we developed an approach to choose an expressive set of basis functions without letting their number grow too large and thereby ensure efficient solving of the minimisation problem later presented in Eq (23). We fit multiple Gaussian mixture models to each time point, varying the number of components until the AIC curve flattened out [29]; in our case this happens at approximately 8 basis functions per time point. To avoid overfitting, we use regularisation to specify minimum diagonal entries of the covariance matrix and encourage separation of basis functions. As our data has been scaled via the commonly used transformation function *f*(*x*) = arcsinh(*x*/5) [30, 31] and then standardised by *z*-scoring, we choose a regularisation value of 1/2; smaller values can be used should one wish to capture sharp peaks, but at the cost of additional basis functions. Finally, for each time point we cluster the data into these Gaussian mixture models and remove poorly populated components. Here, after clustering, we remove any basis functions which represent less than *α* × 100% of the data. Therefore, it should be noted that obtaining a good fit to the matrix *P* is a payoff between: (*i*.) number of basis functions per time point (i.e., what is the maximum number of clusters per time point?); (*ii*.) the regularisation value (i.e., how sharp peaks can one fit?); and (*iii*.) the drop rate *α* (i.e., what fraction of data points does each basis function have to represent?).

**Fig 5:**
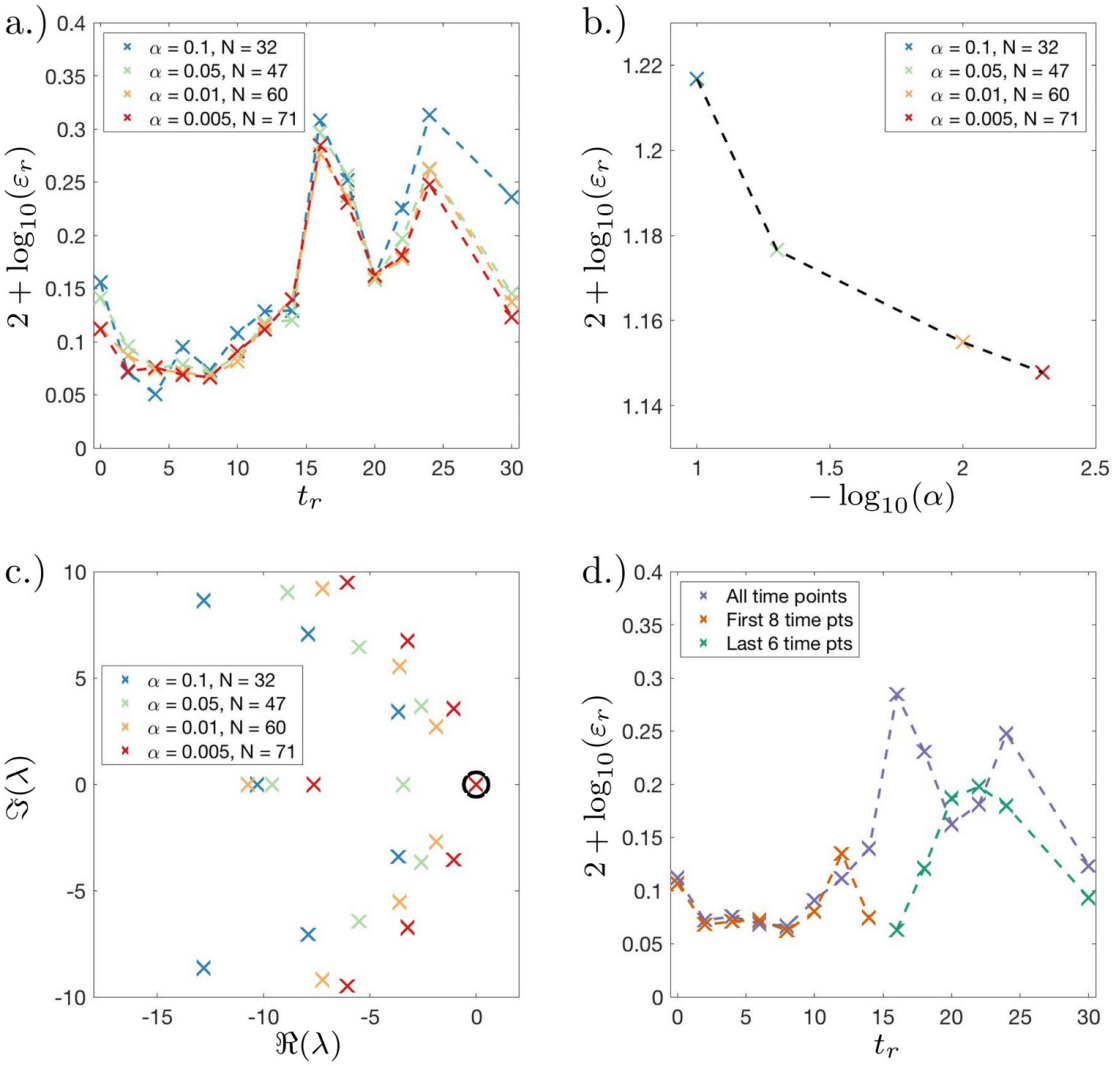
Dynamic distribution decomposition applied to Nanog-Neo cell line data taken from Zunder *et. al*. [28]. (a.) Fitting error plot showing log_10_ transformed percentage error plotted against time for different values of *α* (described in main text); (b.) log-log plot of mean fitting error plotted against −log_10_(*α*), mean error decreases as alpha decreases; (c.) complex plot of eigenvalues λ of Perron–Frobenius matrix *P* for different values of *α*; (d.) Fitting error plot showing log_10_ transformed percentage error plotted against time for *α* = 0.05 (mean error 14.1%), two further Perron–Frobenius matrices are fitted using only the: first 8 time points (mean error 8.4%); and last 6 time points (mean error 14.0%).

We now decrease *α* and evaluate whether we have sufficient basis functions. We plot the percentage fitting error at each time point and the mean percentage error as a function of *α*, see Fig 5(a) and 5(b). These figures show that as *α* decreases, the error only minimally decreases for large increases in the total number of basis functions *N*. We can also view the eigenvalues plotted in the complex plane for various values of *α*, see Fig 5(c). We rescaled time to the unit interval, therefore one will not be able observe eigenfunctions with a corresponding non-zero eigenvalue ℜ(λ) > − 1, i.e., we cannot observe timescales slower than the observation window. We notice for *α* = 0.005 that ℜ(λ_1_) = −1.06 so we are confident decreasing *α* will not offer much benefit. Additionally in the cases where *α* = 0.01 and *α* = 0.005, the extrema of the first few eigenfunctions correspond to the same basis functions (not plotted). For an illustration showing the entries of the coefficient vectors ${\left\{c_{r}\right\}}_{r=1}^{R}$ for the instance when *α* = 0.005, see the S2 Appendix.

#### Loss of dynamic autonomy after stimulus removal

When a stochastic dynamical system is autonomous, the current state of the system determines the likely future states; here we show that after and including *t* = 16 days the system becomes less autonomous, once the Dox induction had ended. We plot the error again at each time point for *α* = 0.005 (mean error 14.1%), see Fig 5(d). We notice that time points after and including *t* = 16 days contain the vast majority of fitting error. To rule out the possibility that the dynamical system instantaneously changed at *t* = 16 days, we fit two Perron–Frobenius matrices, one using the first 8 time points (mean error 8.4%) and a second using the last 6 time points with all fits using the same basis functions (mean error 14.0%). We find that there is still much more error contained in the final 6 time points compared to the first 8.

The autonomous dynamical system assumption means that using the data presented, the future states of the system depend on the current state. While this is likely true within a cell culture system, we only observe a tiny fraction of the state space of the dynamical system as we do not measure the transcriptome and the vast majority of the proteome. Therefore, it seems reasonable to assume that from *t* = 16 days, we are not observing enough of the dynamical system to obtain a linear map between distributions. This insight suggests further single-cell experiments at these later time points using technologies allowing greater ‘omic’ profiling, e.g., single-cell RNA-Seq.

#### Inferred dynamically important states agree with previously described cell subpopulations

**Fig 6:**
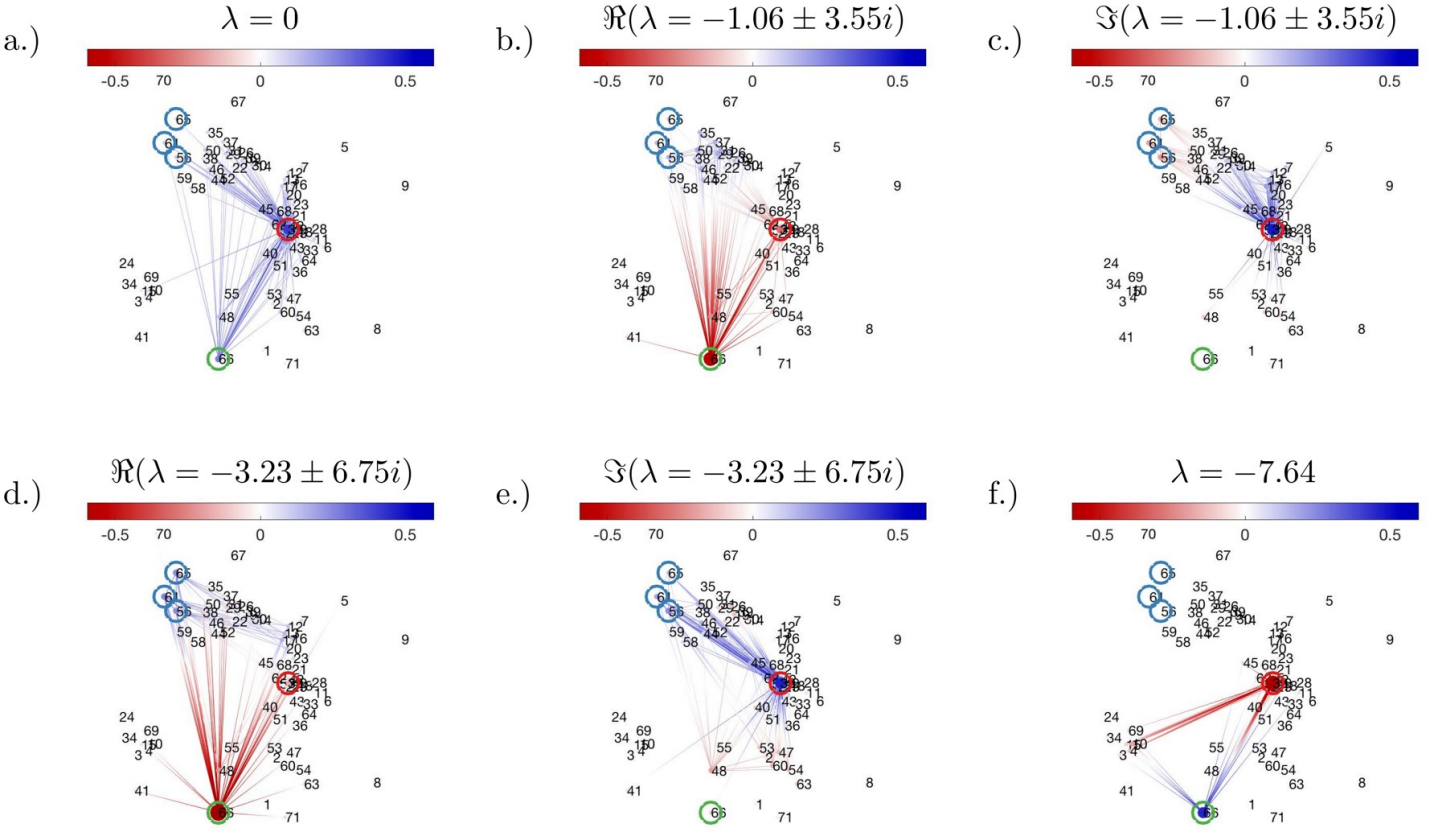
Dynamic distribution decomposition applied to Nanog-Neo cell line data taken from Zunder *et. al*. [28]. Plots (a)–(f) show a graph representation of the eigenfunctions, for full details see Methods section. Groups as defined in the text are circled and coloured in: red (group A, MEF-like); blue (group B, mesendoderm); and green (group C, ESC-like).

We evaluated the extreme of the eigenfunctions of the approximated Perron–Frobenius operator to re-identify cell subpopulations found in Zunder *et. al*. [28]. We first plot the first 6 eigenfunctions, see Fig 6. Nodes that are close together to each other in 18 dimensions (using Euclidean distance) as plotted as close to each other in 2 dimensions. Protein expression of the basis functions are also plotted using the same coordinates as the graph, see extra figure in S2 Appendix.

When examining the extrema of the eigenfunctions, basis functions seem to cluster in 3 groups: group A centred around basis function 32; group B with members 56, 61, and 65; and group C with one member, basis function 66. Our algorithm recovers the same populations as stated in Zunder *et. al*. [28]: cells with low Ki-67 expression do not successfully reprogram and remain MEF-like (group A); cells with high Ki-67 expression then subdivide into two populations, an embryonic stem cell-like (ESC-like) population with Nanog^+^, Sox2^+^, and CD54^+^(group C) and a mesendoderm like population with Nanog^−^, Sox2^−^, Lin-28^+^, CD24^+^expression (group B). As our basis functions were added sequentially per time point, the MEF-like population appeared first.

DDD suggests a few new insights previously not elucidated in Zunder *et. al*. [28]. We find according to the fitted Perron–Frobenius operator, MEF-like cells form the steady state (when λ = 0). Therefore, the model predicts all cells would revert to fibroblasts if enough time passes—although one has to be careful over interpreting predictions due to the higher error after *t* = 16 days.

#### Lasso regularisation reveals sparse topology of iPSC dynamics

**Fig 7:**
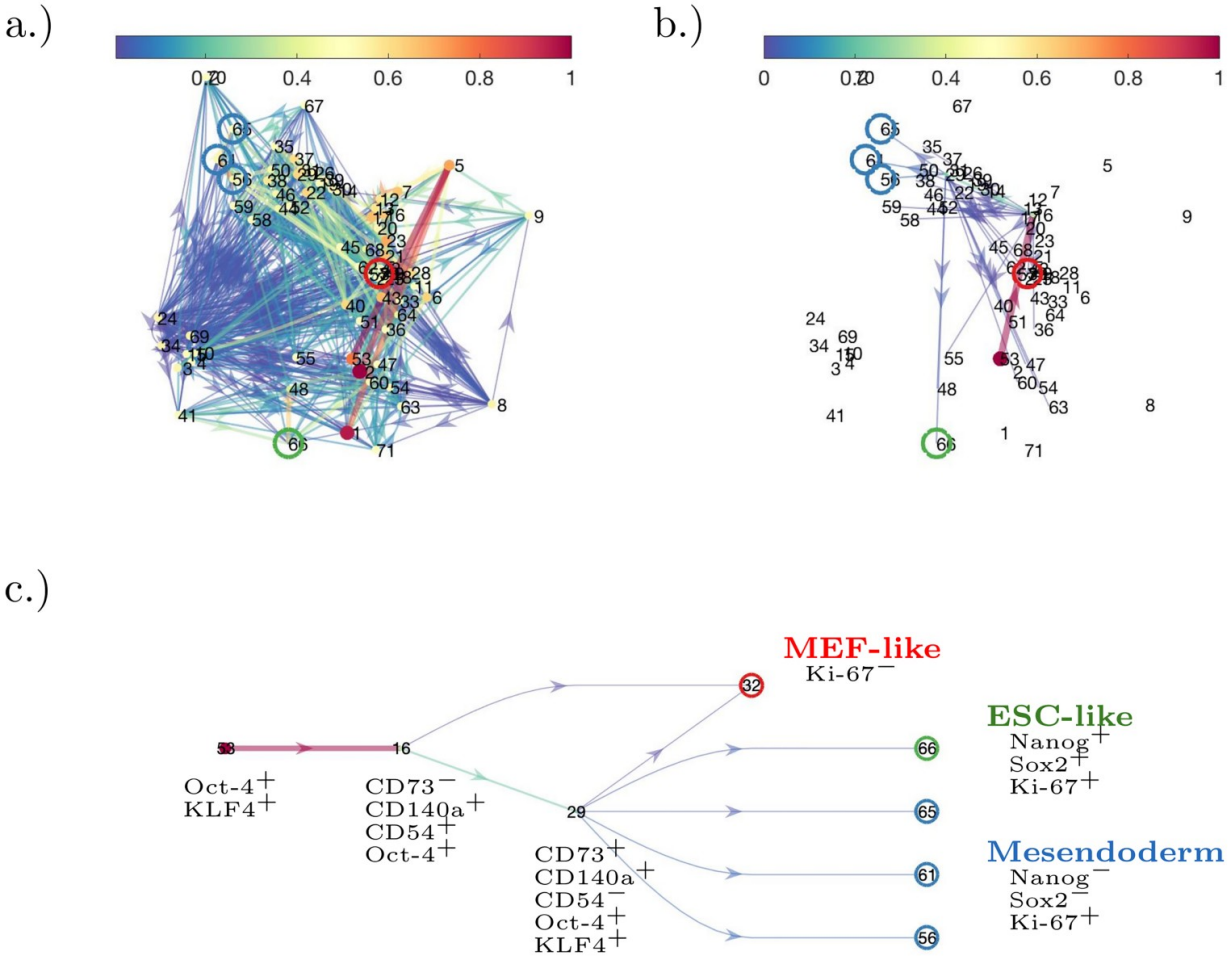
Dynamic distribution decomposition applied to Nanog-Neo cell line data taken from Zunder *et. al*. [28]. The plots show graph representations of *P* (interpreted as a Markov transition rate matrix), colours of edges denote magnitude of rate change from one node to another, and node colours denote rate at which node decays. Colours scaled to unit interval. (a.) matrix *P* plotted without Lasso regularisation; and (b.) matrix *P* plotted with Lasso regularisation, *β* = 1/[800 × mean(*M*)], see Methods section. In (c.) the graph structure from (b.) has unannotated end nodes removed and is rearranged into a simple branching structure. Groups as defined in the text are circled and coloured in: red (group A, MEF-like); blue (group B, mesendoderm); and green (group C, ESC-like).

Reminiscent of the SDE example, the graph as induced by the transition rate matrix *P* is cluttered due to an abundance of low weighted edges, see Fig 7(a); we apply the Lasso modification to reveal a two branching points, see Fig 7(b) and Methods section. Finally, to focus on the 3 groups previously identified, we prune edges leading to unannotated nodes to obtain an easily interpretable branching structure, see Fig 7(c). This figure suggests that at basis function 53 (close to basis function 1, i.e., the initial state), a cell moves to towards branching basis function 16 (CD73^−^, CD140a^+^, CD54^+^, Oct-4^+^), and then has a decision to move towards basis function 32 (group A, MEF-like) or to reach a second branching point at basis function 29 (CD73^+^, CD140a^+^, CD54^−^, Oct-4^+^, KLF4^+^). At basis function 29, the cell will then choose between basis functions 56, 61 and 65 (group B, mesendoderm population), or towards basis function 66 (group C, ESC-like); there is also a weakly weighted edge back to basis function 32 (group A, MEF-like). The state described by basis function 29 was previously described in Zunder *et. al*. [28], but we are able to include an additional transitionary state by means of basis function 16. We can conclude that the cell decision towards becoming remaining MEF-like is made early during the course of the experiment: basis function 16 was placed with the data recorded at 6days; basis function 29 was placed with the data recorded at 12days. As a negative control, the method has also been applied a dataset from Ref. [32] in S4 Appendix.

## Methods

We now give the mathematical set-up to our problem, additional technical details are given in the S1 Appendix, see also [33, 34]. The method follows the following steps: (*i*.) the statement is posed that the temporal evolution of cell states follows a linear partial differential equation; (*ii*.) the distribution of the sample points at each time point can be encoded into a sequence of basis functions; (*iii*.) the weights of these basis functions can change dynamically interpolating between sample points; and (*iv*.) we fit the form of the matrix approximation of differential operator around these changing basis functions; and (*v*.) study the eigenfunctions. The workflow of the method is also as an illustration in Fig 1.

### Mathematical set-up

Assume we have a sequence of *R* experimental readings at time points *t*_1_ < … < *t*_*R*_; without loss of generality we choose *t*_1_ = 0. At each time point, *n*_*r*_ cells are harvested with states $X_{r}=\left\{x_{r,1},\ldots ,x_{r,n_{r}}\right\}$ for *r* = 1, …, *R*. The state of each cell is located in a (measurable) space, x∈M. We note that this space may not be the full dimension of the data set, but after a dimensionality reduction technique has been applied, e.g., PCA, diffusion maps etc. For example, in the case of RNA-Seq data, the state of a cell would consist of thousands of genes which would be too high to apply kernels to. We wish to find a probability distribution *ϱ* = *ϱ*(*t*, ***x***) such that when *t* = *t*_*r*_, the probability of observing cells in states ***X***_*r*_ would be highly probable for *r* = 1, …, *R*.

Immediately necessary to ensure conservation of mass, we require

$$\begin{array}{c} \int_{M}\varrho \left(t,x\right)\text{d}x=1,\;\text{and}\;\varrho \left(t,x\right)\geq 0, \end{array}$$ (3)

for all *t* ∈ (*t*_1_, *t*_*R*_), i.e., *ϱ* is a probability density function (PDF). We now make the crucial assumption that our method relies on: each cell follows a (well behaved) autonomous dynamical system—implicit in this assumption is that cells do not interact, alternatively cell interactions can be accounted for via stochastic noise terms. Under these assumptions, we can interpret *ϱ*(*t*, ***x***)Δ***x*** as the probability a randomly selected cell has state in the interval [***x***, ***x*** + Δ ***x***) at time *t*. We write down the (continuous-time) Perron–Frobenius equation for the dynamics of the density profile as

$$\begin{array}{c} \frac{\partial }{\partial t}\varrho \left(t,x\right)=P\varrho \left(t,x\right)\;, \end{array}$$ (4)

for initial condition *ϱ*(*t* = 0, ***x***) = *ϱ*_0_(***x***). The P term is the continuous-time Perron–Frobenius operator [21, 22]. The Perron–Frobenius operator associated with a dynamical system maps a density on the state space to another density on the state space. To build intuition for P, consider a discrete-time Markov chain over a countable number of discrete states ***x***_*r*_ with recursive relation

$$\begin{array}{c} \varrho \left(\left(m+1\right)\Delta t,x_{r}\right)=\sum_{s}k_{\Delta t}\left(x_{r}|x_{s}\right)\varrho \left(m\Delta t,x_{s}\right)\;,\;m=0,1,2,\ldots \;, \end{array}$$ (5)

where *k*_Δ*t*_(***x***_*r*_|***x***_*s*_) is a kernel that specifies the probability that state ***x***_*s*_ is mapped to state ***x***_*r*_ and therefore ∑_*r*_ *k*_Δ*t*_(***x***_*r*_|***x***_*s*_) = 1. Moving from a discrete space to our continuous state space M (by defining and taking limits appropriately), the summation in Eq (5) becomes the integral

$$\begin{array}{c} \varrho \left(\left(m+1\right)\Delta t,x\right)=\int_{M}k_{\Delta t}\left(x|y\right)\varrho \left(m\Delta t,y\right)\text{d}y\;\;,\;m=0,1,2,\ldots \;. \end{array}$$ (6)

Returning to Eq (4), one can exponentiate the operator P and we can thus relate the transition density function *k*_Δ*t*_ in Eq (6) to the operator P via the relation

$$\begin{array}{c} e^{\Delta tP}\varrho \left(m\Delta t,x\right)=\int_{M}k_{\Delta t}\left(x,y\right)\varrho \left(m\Delta t,y\right)\text{d}y\;. \end{array}$$ (7)

Therefore, the operator $e^{\Delta tP}$ maps the distribution of states at one time point to a new distribution Δ*t* > 0 units of time later. The operator P is known by many names depending on the underlying dynamical system for the state evolution x(t)∈M for *t* ∈ (*t*_1_, *t*_*R*_). For example, within SDEs Eq (4) is a second order parabolic PDE known as the Fokker–Planck equation [35]; and for chemical reaction networks Eq (4) is a system of coupled ODEs known as the chemical master equation [36].

### Finite dimensional approximation

We would like to find a finite dimensional approximation of of P; we can do this with non-negative basis functions ***ψ***(***x***) = [*ψ*_1_(***x***), …, *ψ*_*N*_(***x***)]^⊺^. We take the ansatz that for all *t* ∈ (*t*_1_, *t*_*R*_), we can expand *ϱ* as the linear combination

$$\begin{array}{c} \tilde{\varrho }\left(t,x\right)=c^{⊺}\left(t\right)\psi \left(x\right)=\sum_{j=1}^{N}c_{j}\left(t\right)\psi_{j}\left(x\right)\;, \end{array}$$ (8)

where ***c***(*t*) = [*c*_1_(*t*), …, *c*_*N*_(*t*)]^⊺^ and we use a tilde (∼) over *ϱ* to denote this approximation. Eq (8) is sometimes known as a Galerkin approximation and is one of the key steps in deriving finite element method numerical schemes to solve partial differential equations. To ensure the probability density integrates to one, we require ***c*** ∈ Λ where

$$\begin{array}{c} \Lambda =\{c\in R^{N}:c^{⊺}\omega =1\;\text{and}\;c^{⊺}\psi \left(x\right)\geq 0\}\;\text{for}\;\omega ={\left[\omega_{1},\ldots ,\omega_{N}\right]}^{⊺}\;\text{and}\;\omega_{j}=\int_{M}\psi_{j}\left(x\right)\text{d}x\;. \end{array}$$ (9)

If the basis functions are themselves probability density functions, then Λ is the probability simplex. In Fig 1(a), we show how a distribution can be represented as a sum of normal distributions, that is, the density is given by a Gaussian mixture model.

We can derive a linear system of ODEs for the coefficients ***c***(*t*). We do this by noting in weak form [37, 38] Eq (4) is

$$\begin{array}{c} {\left\langle g,\dot{\varrho }\right\rangle }_{M}={\left\langle g,P\varrho \right\rangle }_{M}\;\text{for}\;{\left\langle g,\varrho \right\rangle }_{M}=\int_{M}g\varrho \;\text{d}x\;. \end{array}$$ (10)

Choosing *g* = *ψ*_*i*_ and expanding *ϱ* as in Eq (8), then

$$\begin{array}{c} M\dot{c}\left(t\right)=Qc\left(t\right)\;, \end{array}$$ (11)

where *M*_*ij*_ = 〈*ψ*_*i*_, *ψ*_*j*_〉 and $Q_{ij}=\langle \psi_{i},P\psi_{j}\rangle$. Assuming *M* is invertible, define

$$\begin{array}{c} P≔M^{-1}Q\;, \end{array}$$ (12)

which is the projection of P onto the basis functions {*ψ*_1_, …, *ψ*_*N*_}. That is, for *g*(***x***) = ***c***^⊺^*ψ*(***x***), we have the equality $Pg\left(x\right)={\left(Pc\right)}^{⊺}\psi \left(x\right)$. In order to preserve probability density and positivity, we require P∈P for

$$\begin{array}{c} P=\{P\in R^{N\times N}:\sum_{k=1}^{N}\omega_{k}P_{kj}=0\;\;\text{and}\;\;P_{ij}\geq 0\;\text{for}\;i,j=1,\ldots ,N\;\text{and}\;i\neq j\}\;. \end{array}$$ (13)

The explanation behind Eq (13) is contained in the S1 Appendix.

We can solve the dynamics for Eq (4) using the approximation in Eq (8) by using the matrix exponential operation, specifically

$$\begin{array}{c} c\left(t\right)=e^{tP}c_{*}\;, \end{array}$$ (14)

where $c_{*}$ are the coefficients at corresponding to the chosen initial condition ${\tilde{\varrho }}_{0}=c_{*}^{⊺}\psi \left(x\right)$. To prevent numerical instability for large times, we rescale the experimental time course (*t*_1_, *t*_*R*_) to the unit interval.

### Calculation of Galerkin approximation coefficients for PDFs

There are many option for selecting basis functions, later discussed in the Conclusion. One option is to use probability density functions, so

$$\begin{array}{c} \int_{M}\psi_{j}\left(x\right)\text{d}x=1\;, \end{array}$$ (15)

for *j* = 1, …, *N*. We can find the values of ***c***_*r*_ at the observed time points by noting that the value of the coefficient at time *t* = *t*_*r*_ must be proportional to the probability that basis function *j* created the data at that time point, so

$$\begin{array}{c} c_{j}\left(t_{r}\right)\propto \sum_{i=1}^{n_{r}}\psi_{j}\left(x_{r,i}\right)\;. \end{array}$$ (16)

One can then normalise $\sum_{j=1}^{N}c_{j}=1$ for *j* = 1, …, *N* to find the coeffient vector ***c***_*r*_.

It is worth remarking here that entries in the coefficient vectors ${\left\{c_{r}\right\}}_{r=1}^{R}$ may change should the data be perturbed. Additionally, depending on the choice in basis functions, the basis functions ${\left\{\psi_{j}\right\}}_{j=1}^{N}$ *may* depend on the data, e.g., when the placement of the Gaussians basis functions is done via the expectation maximisation algorithm for Gaussian mixture models. In the event that one uses global basis functions, e.g., orthogonal polynomials, the basis are chosen independent of the data, and therefore perturbations to the data would only affect ${\left\{c_{r}\right\}}_{r=1}^{R}$ but not the form of the basis functions themselves.

### Selection of *P* matrix

We now need to address the issue of how to determine *P* from data. We now motivate a choice in error to minimise, generally analogous to least squares fitting error for ODEs.

We are interested in how an initial state goes on to predict later recorded states. We do not use the first calculated coefficient, ***c***_1_, as the initial condition, but allow for mis-specification of the initial condition by specifying that it is a free parameter to the model. The initial condition is given as density $\varrho_{0}=\varrho_{0}\left(x\right)$ with Galerkin approximation

$$\begin{array}{c} {\tilde{\varrho }}_{0}=c_{*}^{⊺}\psi \left(x\right)\;. \end{array}$$ (17)

Consider a linear operator P with matrix representation *P* on the space spanned by ***ψ***. The *L*^2^ norm gives a measure of how well P represents the evolution of the densities. The squared relative prediction error at time *t* = *t*_*r*_ for *r* = 1, …, *R* is

$$\begin{array}{cc} \epsilon_{r}^{2}\left(P,\varrho_{0}\right) & ≔\frac{∥e^{t_{r}P}\varrho_{0}\left(\cdot \right)-\varrho \left(t_{r},\cdot \right){∥}_{L^{2}}^{2}}{∥\varrho \left(t_{r},\cdot \right){∥}_{L^{2}}^{2}}=\frac{\int_{M}{\left[e^{t_{r}P}\varrho_{0}\left(x\right)-\varrho \left(t_{r},x\right)\right]}^{2}\text{d}x}{\int_{M}{\left[\varrho (t_{r},x)\right]}^{2}\text{d}x}\;. \end{array}$$ (18)

Notice in Eq (18) that the error is a function of both the Perron–Frobenius operator and the initial condition. We then define the mean squared relative prediction error as

$$\begin{array}{cc} \epsilon^{2}\left(P,\varrho_{0}\right) & =\frac{1}{R}\sum_{r=1}^{R}\epsilon_{r}^{2}\left(P,\varrho_{0}\right)\;. \end{array}$$ (19)

It would be ideal now to find

$$\begin{array}{c} \left\{P,\varrho_{0}\right\}≔\underset{P,\;\varrho_{0}\geq 0,\varrho_{0}\in L^{1}}{arg min}\epsilon^{2}\left(P,\varrho_{0}\right)=\underset{P,\;\varrho_{0}\geq 0,\varrho_{0}\in L^{1}}{arg min}\frac{1}{R}\sum_{r=1}^{R}\epsilon_{r}^{2}\left(P,\varrho_{0}\right)\;. \end{array}$$ (20)

Of course, we do not know what this error is without using our finite dimensional Galerkin approximation; therefore we approximate

$$\begin{array}{c} \epsilon_{r}^{2}\left(P,\varrho_{0}\right)\overset{\text{Eqs.}\;\text{(}8\text{,}\;17\text{)}\;}{\approx }\varepsilon_{r}^{2}\left(P,c_{*}\right)\;. \end{array}$$ (21)

and we calculate the time *t* = *t*_*r*_ error as

$$\begin{array}{c} \varepsilon_{r}^{2}\left(P,c_{*}\right)≔\frac{∥{\left(e^{t_{r}P}c_{*}-c_{r}\right)}^{⊺}\psi \left(\cdot \right){∥}_{L^{2}}^{2}}{∥c_{r}^{⊺}{\psi \left(x\right)∥}_{L^{2}}^{2}}=\frac{∥e^{t_{r}P}c_{*}-c_{r}{∥}_{M}^{2}}{∥c_{r}{∥}_{M}^{2}}=\frac{{\left[e^{t_{r}P}c_{*}-c_{r}\right]}^{⊺}M\left[e^{t_{r}P}c_{*}-c_{r}\right]}{c_{r}^{⊺}Mc_{r}}\;, \end{array}$$ (22)

where we introduced the norm weighted by the mass matrix ${∥c∥}_{M}≔\sqrt{c^{⊺}Mc}$. Our objective function is modified by using this finite dimensional approximation to

$$\begin{array}{c} \left\{P,c_{*}\right\}≔\underset{P\in P,\;c_{*}\in \Lambda }{arg min}\varepsilon^{2}\left(P,c_{*}\right)=\underset{P\in P,\;c_{*}\in \Lambda }{arg min}\frac{1}{R}\sum_{r=1}^{R}\varepsilon_{r}^{2}\left(P,c_{*}\right)\;. \end{array}$$ (23)

**Algorithm 1** Algorithm to Determine Perron-Frobenius Matrix

**Require**: Data $X={\left\{X_{r}\right\}}_{r=1}^{R}$ and observation times $t_{1}<\cdots <t_{R}$.

**Require**: Choose basis functions $\psi (x)=[\psi_{1}(x),\ldots ,\psi_{N}(x){]}^{⊺}$.

1: Solve

$$\left\{P,c_{*}\right\}≔\underset{P\in P,c_{*}\in \Lambda }{arg min}\frac{1}{R}\sum_{r=1}^{R}\varepsilon_{r}^{2}\left(P,c_{*}\right).$$

2: **return** P

Unmentioned at this point is that: for a large quantity of basis functions the size over which this optimisation problem occurs is challenging. That is: one has *N*^2^ free elements in the matrix *P*, with zero column sums one has *N*(*N* − 1) degrees of freedom; but one also has the initial condition to choose adding *N* parameters, or *N* − 1 degrees of freedom (with unit column sum)—in total (*N* − 1)(*N* + 1) degrees of freedom. Therefore, the problem is unapproachable without gradient calculations to speed up the optimization algorithm. Using the exponential matrix derivative (see S1 Appendix), one can calculate the *t* = *t*_*r*_ relative error with respect to *P* as

$$\frac{\partial \varepsilon_{r}^{2}}{\partial P}=\frac{2}{c_{r}^{⊺}Mc_{r}}\sum_{k=0}^{\infty }\frac{t^{k+1}}{(k+1)!}\sum_{j=0}^{k}{\left(P^{k-j}\right)}^{⊺}M\left(e^{t_{r}P}c_{*}-c_{r}\right)c_{*}^{⊺}{\left(P^{j}\right)}^{⊺}$$ (24)

$$=\frac{2}{c_{r}^{⊺}Mc_{r}}\sum_{k=0}^{\infty }\frac{t^{k+1}}{(k+1)!}S_{k}\;,$$ (25)

and the derivative with respect to c_*_ as

$$\begin{array}{c} \frac{\partial \varepsilon_{r}^{2}}{\partial c_{*}}=\frac{2}{c_{r}^{⊺}Mc_{r}}{\left[e^{t_{r}P}\right]}^{⊺}M\left[e^{t_{r}P}c_{*}-c_{r}\right]\;. \end{array}$$ (26)

The terms

$$\begin{array}{c} S_{k}=\sum_{j=0}^{k}{\left(P^{k-j}\right)}^{⊺}M\left(e^{t_{r}P}c_{*}-c_{r}\right)c_{*}^{⊺}{\left(P^{j}\right)}^{⊺}\; \end{array}$$ (27)

can be calculated using the recursion relation

$$\begin{array}{c} S_{k}=P^{⊺}S_{k-1}+S_{k-1}P^{⊺}-P^{⊺}S_{k-2}P^{⊺}\;\text{where}\;S_{-1}=0\;\text{and}\;S_{0}=M\left(e^{t_{r}P}c_{*}-c_{r}\right)c_{*}^{⊺}\;. \end{array}$$ (28)

#### Lasso regularisation

For display purposes, we can promote sparsity in *P* by using a Lasso regularisation. We choose the regularisation parameter such that there is at least a single edge connected to the extrema of the first few eigenfunctions. We modify the error term in Eq (23) to

$$\begin{array}{c} \varepsilon_{†}^{2}\left(P,c_{*}\right)=\frac{1}{R}\sum_{r=1}^{R}\varepsilon_{r}^{2}\left(P,c_{*}\right)+\beta ∥\text{vec}\left(M○P\right){∥}_{1}=\frac{1}{R}\sum_{r=1}^{R}\varepsilon_{r}^{2}\left(P,c_{*}\right)+\beta \sum_{i=1}^{N}\sum_{j=1}^{N}\left|M_{i,j}P_{i,j}\right|\;, \end{array}$$ (29)

where ○ denotes the Hadamard product (or entrywise product) and vec(⋅) denotes the vectorisation of a matrix. In the case where the basis functions are probability density functions, we can calculate the derivative of this expression as

$$\begin{array}{c} \frac{\partial }{\partial P}({∥\text{vec}\left(M○P\right)∥}_{1})=[\begin{array}{cccc} -M_{1,1} & M_{1,2} & \cdots & M_{1,N}\\ M_{2,1} & -M_{2,2} & & \vdots \\ \vdots & & \ddots & \\ M_{N,1} & \cdots & & -M_{N,N} \end{array}]\;. \end{array}$$ (30)

By using the mass matrix *M* as a weighting in front of the Perron–Frobenius matrix *P*, we are promoting edges between basis functions located apart from each other.

### Graph visualisations

#### *P* matrix visualisations

One can interpret the matrix *P* as a Markov transition rate matrix, in which case the entry *P*_*i*,*j*_ shows the rate at which state *j* transitions into state *i*. Therefore, a cell in cluster *i* will switch to cluster *j* in time interval [*t*, *t* + Δ*t*) for Δ*t* > 0 with probability $P_{i,j}\Delta t+O\left(\Delta t^{2}\right)$. To reiterate, cells exist in states between the basis functions so instead of being at a single state, they are in a state which is a weighted combination of the basis functions. However, this interpretation allows us to plot a directed network with weighted adjacency matrix *P*_*i*,*j*_. Nodes can then be placed using one of a multitude of algorithms, in our case we use force-directed node placement with weights inversely proportional to the mass matrix *M*. Visualisations of the mass matrices associated to the problems in this manuscript are contained in the S2 Appendix. We also plot the size of node *i* proportional to *P*_*i*,*i*_ (as this is the rate at which state *i* remains in state *i*).

#### Eigenfunction visualisation

To investigate key dynamical behaviours of a linear operator, a common theme is the study of the corresponding eigenproblem. By solving the eigenproblem, one can decompose the solution of the operator into components (known as eigenfunctions or eigenvectors) that will dynamically change with respect to the eigenvalue. By studying the eigenproblem, one can break down the solution into key behaviours and find important transitionary states.

For an eigenfunction satisfying

$$\begin{array}{c} P\varrho_{\lambda }\left(x\right)=\lambda \varrho_{\lambda }\left(x\right)\;, \end{array}$$ (31)

using the finite dimensional Galerkin approximation in Eq (8), there is the corresponding eigenvector

$$\begin{array}{c} Pv_{\lambda }=\lambda v_{\lambda }\;. \end{array}$$ (32)

In low dimensions, one can simply plot the original function *ϱ*_λ_ as the linear combination of basis functions

$$\begin{array}{c} {\tilde{\varrho }}_{\lambda }\left(x\right)=v_{\lambda }^{⊺}\psi \left(x\right)=\sum_{j=1}^{N}v_{\lambda ,j}\psi_{j}\left(x\right)\;, \end{array}$$ (33)

To ensure consistent scales when plotting, we demand

$$\begin{array}{c} ∥\varrho_{\lambda }{∥}_{L^{2}}={\left\langle \varrho_{\lambda },\varrho_{\lambda }\right\rangle }_{M}=1\;, \end{array}$$ (34)

or when considering the finite dimensional Galerkin approximation

$$\begin{array}{c} ∥{\tilde{\varrho }}_{\lambda }{∥}_{L^{2}}=∥v_{\lambda }^{⊺}{\psi \left(\cdot \right)∥}_{L^{2}}={∥v_{\lambda }∥}_{M}={\left\langle v_{\lambda },v_{\lambda }\right\rangle }_{M}=v_{\lambda }^{⊺}Mv_{\lambda }={\left\langle M^{\frac{1}{2}}v_{\lambda },M^{\frac{1}{2}}v_{\lambda }\right\rangle }_{2}=1\;. \end{array}$$ (35)

In high dimensions, our ability to visualise functions is limited. However, we have represented the function as a linear combination of basis functions; one option for visualisation purposes is to present the coefficients in the eigenvector. To visualise if the eigenvector values have similar or dissimilar values, we can consider representing the eigenfunction as a graph. We specify the adjacency matrix for an undirected weighted graph as the outer product

$$\begin{array}{c} G_{\lambda }=\left(M^{\frac{1}{2}}v_{\lambda }\right)⊗\left(M^{\frac{1}{2}}v_{\lambda }\right)=M^{\frac{1}{2}}v_{\lambda }v_{\lambda }^{⊺}M^{\frac{1}{2}}\;. \end{array}$$ (36)

For Perron–Frobenius eigenfunctions with eigenvalue λ ≠ 0, the function will obtain both a positive and negative values (indicating where probability mass is flowing from and to). Therefore, by examining *G*_λ_, a positive value in entry (*i*, *j*) in *G*_λ_ indicates basis functions *i* and *j* both have the same sign (positive or negative) and a negative value indicates they have opposite signs. By plotting edges as straight lines with thickness proportional to the values in *G*_λ_, we can visualise pairs of nodes (positioned in high dimensional space) and show an approximate gradients in between these nodes. When entries of *G*_λ_ are small, then only very thin lines will be plotted between nodes, indicating the lack of connection between these areas in state space.

By using the weighting of $M^{\frac{1}{2}}$ in front of the eigenvector ***v***_λ_ we ensure that the sizes of the eigenvectors are bounded. Occasionally it is the case that we get complex eigenvalues, in which case they appear as complex conjugates and one can plot the real and imaginary parts separately.

### Conclusion

We presented Dynamic Distribution Decomposition for identification of dynamically important states of biological processes. This method operates on snapshot time series data and infers dynamically important states by mapping between distributions. By fitting a very general autonomous dynamical system over basis functions encoding the observed raw dataset, one is able to obtain fitting error estimates indicating said level of autonomy within the dataset. We applied our approach to synthetic data generated for a simple test system, and then further to a mass cytometry time series data set for iPSC reprogramming. Our approach performed well for both systems showing key dynamical states. For the experimental system of iPSC reprogramming of fibroblasts, we could also identify key time points (with large fitting error) where the current experimental (or computational) set-up is insufficient to elucidate the reprogramming process. This suggests one of several reasons, either 1.) limitations of the model: non-autonomy (e.g., delayed models more appropriate, cell interactions important), poor selection of basis functions; or 2.) limitations of the data: selection of uninformative measured genes/proteins, or measurement error. Due to our careful approach in selecting basis functions, we believe further experimental investigation is warranted.

DDD can be computed efficiently, e.g., via the recursion relation given by Eq (28), but we have not optimised the implementation. In the case where there is more than 100 basis functions, our minimisation procedure using the inbuilt MATLAB multivariate minimisation algorithm can be unreasonably slow; therefore, we will investigate more efficient implementations.

DDD depends on a few design decisions, such as the choice of basis functions. In this manuscript, we used basis functions as components of a Gaussian mixture model and gave parameters that needed tuning to alter the fit. Our method for choosing basis functions does not have an optimal configuration with regards to minimising error. This is because by sending the regularisation value to infinity one obtains perfect fits, and sending the regularisation value to zero can lead to ill fitting solution (basis functions with same mean etc). However, one can use the *α* parameter as a rule of thumb to ensure enough basis functions are included. For other applications other choices in basis functions are conceivable, for example, radial basis functions [39]; piecewise linear basis functions [40]; and global basis functions [21, 27] to name a few. When the basis functions have finite support, the mass matrix *M* will be sparse, in which case the Lasso step will not be necessary. It is worth noting that calculation of the mass matrix analytically is only possible in a few cases and Monte–Carlo integration may be necessary—which would add an additional source of error to the methodology. Practically, it would also make sense to use basis functions built around the data type, for example negative binomial distributions are often used to model UMI counts from single-cell RNA-Seq data. When one uses a single basis function centred around each data point, one refers to this as kernel density estimation, of which there are optimal methods to choose the basis function [41]; when using a small number of basis functions for a large number of sample points, there are likely optimal ways of choosing them which we will investigate in future work.

DDD could be applied to investigate pseudotime ordered single-cell data of single time point experiments, see [42, 43] and S3 Appendix. Here, one uses single-cell data measured at only a single time point to carry out trajectory inference and subpopulation identification to infer biological processes, e.g., the cell cycle; reviews of such methods can be found in Ref. [44, 45]. It may be possible to improve our fits by combining approaches: while cells are monitored with regards to experimental time, individual cell time coordinates might deviate due to asychronity of process initiation; this could be incorporated to get smoother Perron–Frobenius operators between time points, see Ref. [46]. This would then be a biologically motivated method to account for delays in the system.

DDD is applicable to evaluate the outcome of reconstruction approaches yielding potential functions. An example of such an approach is reported by Hashimoto et. al. [47], where hypothesised potential function is reconstructed under the following assumptions: *i.)* the data was generated by SDEs in the potential well; and *ii.)* the gradient of the potential is a single layer of a sigmoid neural network. Our reported eigen-decompositions is applicable to identify dynamically important states for the inferred potential function.

The work presents Dynamic Distribution Decomposition, linking advances in operator theory to applied practice in high-dimensional data analysis. While we focus on application of DDD to mass cytometry measurements, it is conceivable to expand to applications to single-cell RNA sequencing time series as well as biological processes other than an iPSC reprogramming. We expect DDD and method variations will be instrumental in providing intuitive understanding of such biological processes.

## Supporting information

S1 Appendix Additional mathematical details. A document detailing the additional mathematical details regarding: the inner products between multivariate normal distributions; requirements on the Perron–Frobenius matrix approximation; a full derivation of the gradient calculation; and additional evidence regarding detection of non-autonomy.
(PDF)

S2 Appendix Additional figures. A document containing two additional figures: protein expression of Nanog-Neo cell line data taken from Zunder *et. al*.; and plots containing visualisations of the coefficient vectors ***c***_*r*_ and mass matrix *M* for both the the stochastic dynamical system and Nanog-Neo cell line data.
(PDF)

S3 Appendix Comparison with other established methods. A document detailing a brief comparison between our method and 3 other published methods: Wishbone [42], Monocle2 [43], and Waddington-OT [6].
(PDF)

S4 Appendix Reconstruction of EGFR signalling from mass cytometry time series data. A document detailing DDD applied to an additional control dataset with known underlying cellular behaviour, see Lun *et. al*. [32].
(PDF)
