# Supplementary material for: Dynamic distribution decomposition for single-cell snapshot time series identifies subpopulations and trajectories during iPSC reprogramming
Source: PLoS Comput Biol. 2020 Jan 10;16(1):e1007491. doi: 10.1371/journal.pcbi.1007491 (PMC6953770; doi:10.1371/journal.pcbi.1007491)
Supplement: S1 Appendix — A document detailing the additional mathematical details regarding: the inner products between multivariate normal distributions; requirements on the Perron–Frobenius matrix approximation; a full derivation of the gradient calculation; and additional evidence regarding detection of non-autonomy. (PDF) [file pcbi.1007491.s001.pdf]

S1 Appendix: Additional Mathematical Details

Inner Products between Multivariate Normal Distributions

In this work, we only used multivariate Normal distributions. Conveniently, expressions exist for the inner product between two Normal distributions. Assuming multivariate Normal distributions as basis functions, we have

$$\psi_j(\boldsymbol{x}) = \mathcal{N}(\boldsymbol{x}\,; \boldsymbol{\mu}_j, \Sigma_j)\,, \tag{37}$$

where $\mathcal{N}$ is the probability density function for a multivariate Normal distribution with mean $\boldsymbol{\mu}$ and covariance matrix Σ. To determine the mass matrix, adapting results from Ref. [33] we can analytically calculate the inner product between two multivariate normal distributions as

$$\langle \psi_1, \psi_2 \rangle_{\mathcal{M}} = \int_{\mathcal{M}} \mathcal{N}(\boldsymbol{x}\,; \boldsymbol{\mu}_1, \Sigma_1)\mathcal{N}(\boldsymbol{x}\,; \boldsymbol{\mu}_2, \Sigma_2)\mathrm{d}\boldsymbol{x} \tag{38}$$

$$= \frac{\exp\left\{\frac{1}{2}\left[\boldsymbol{\eta}_1^{\mathsf{T}}\Sigma_1\boldsymbol{\eta}_1 + \boldsymbol{\eta}_2^{\mathsf{T}}\Sigma_2\boldsymbol{\eta}_2 - \boldsymbol{\eta}_*^{\mathsf{T}}(\Sigma_1^{-1} + \Sigma_2^{-1})^{-1}\boldsymbol{\eta}_*\right]\right\}}{\sqrt{(2\pi)^d|\Sigma_1|\,|\Sigma_2|\,|\Sigma_1^{-1} + \Sigma_2^{-1}|}}\,, \tag{39}$$

for

$$\boldsymbol{\eta}_1 = \Sigma_1^{-1}\boldsymbol{\mu}_1\,, \quad \boldsymbol{\eta}_2 = \Sigma_2^{-1}\boldsymbol{\mu}_2\,, \quad \text{and} \quad \boldsymbol{\eta}_* = \boldsymbol{\eta}_1 + \boldsymbol{\eta}_2\,. \tag{40}$$

Requirements on Perron–Frobenius Matrix Approximation

To preserve probability, it must be the case that

$$\sum_{i=1}^{N} \omega_i P_{ij} = 0 \quad \forall j = 1, \ldots, N\,, \tag{41}$$

which is identical to the condition for continuous time Markov chains. This is because we require

$$\frac{\mathrm{d}}{\mathrm{d}t}\int_{\mathcal{M}} \varrho(t, \boldsymbol{x})\mathrm{d}\boldsymbol{x} = 0 \tag{42}$$

$$= \frac{\mathrm{d}}{\mathrm{d}t}\int_{\mathcal{M}} \boldsymbol{c}^{\mathsf{T}}(t)\boldsymbol{\psi}(\boldsymbol{x})\mathrm{d}\boldsymbol{x} \tag{43}$$

$$= \int_{\mathcal{M}} \dot{\boldsymbol{c}}^{\mathsf{T}}(t)\boldsymbol{\psi}(\boldsymbol{x})\mathrm{d}\boldsymbol{x} \tag{44}$$

$$= \dot{\boldsymbol{c}}^{\mathsf{T}}(t)\boldsymbol{\omega} = \boldsymbol{\omega}^{\mathsf{T}}\dot{\boldsymbol{c}}(t) \tag{45}$$

$$= \boldsymbol{\omega}^{\mathsf{T}}P\boldsymbol{c}(t) \tag{46}$$

$$= \sum_{i=1}^{N} \omega_i \left[\sum_{j=1}^{N} P_{ij}c_j(t)\right] \tag{47}$$

$$= \sum_{j=1}^{N} c_j(t) \left(\sum_{i=1}^{N} \omega_i P_{ij}\right) \tag{48}$$

Therefore, for mass to be conserved, equation (13) holds.

We also require the operator $e^{t\mathcal{P}}$ preserves positivity, that is, $e^{t\mathcal{P}}u_0 \geq 0$ for functions $u_0 \geq 0$. This implies that the off-diagonal entries of P must be non-negative. The reason is as follows. As $\boldsymbol{\psi} \geq 0$, the function

$u_0 = c_u^\intercal \boldsymbol{\psi}$ must have $c_u \geq 0$ due to linear independence of the normalised basis functions. It therefore follows that $e^{tP} \geq 0$ element wise, because the positivity condition and linear independence of the normalised basis functions require that $c_u^\intercal e^{tP} \geq 0$ element wise. Thus, $0 \leq e^{tP} = I + tP + \mathcal{O}(t^2)$ for all $t \geq 0$, which in particular means that $tP_{i,j} + \mathcal{O}(t^2) \geq 0$ for $i \neq j$. The condition $P_{i,j} \geq 0$ follows by considering arbitrarily small $t > 0$. This is sufficient for e^{tP} to be element wise non-negative, as the matrix exponential of a matrix with non-negative off-diagonal elements is always non-negative element wise. Note that this, together with the mass conservation condition, implies that the diagonal entries of P are all negative.

Gradient Calculation

For relevant background reading on functions of matrices, see Ref. [34]. Let $\mathcal{H}$ denote the Hilbert space $\mathbb{R}^N$ with inner product $\langle \boldsymbol{p}, \boldsymbol{q} \rangle_{\mathcal{H}} = \boldsymbol{p}^\intercal M \boldsymbol{q}$, $P \in \mathbb{R}^{N\times N}$. For $t > 0$, and $\boldsymbol{p}, \boldsymbol{q} \in \mathcal{H}$, we derive the gradient of $f(P) = \|e^{tP}\boldsymbol{q} - \boldsymbol{p}\|_{\mathcal{H}}^2$ that is used for the gradient calculation in Equation (24). Let $D_f(P; dP)$ be the directional derivative of f at P, in the direction dP. Let $\langle \cdot, \cdot \rangle_F$ denote the Frobenius inner product on $\mathbb{R}^{N\times N}$. The gradient of f is the matrix $G \in \mathbb{R}^{N\times N}$ such that $D_f(P; dP) = \langle G, dP \rangle_F$ for any $dP \in \mathbb{R}^{N\times N}$.

First, note that

$$D_f(P; dP) = 2(e^{tP}\boldsymbol{q} - \boldsymbol{p})^\intercal M[D_{e^{t\cdot}}(P, dP)]\boldsymbol{q} = 2\langle e^{tP}\boldsymbol{q} - \boldsymbol{p}, [D_{e^{t\cdot}}(P, dP)]\boldsymbol{q}\rangle_{\mathcal{H}}\,. \tag{49}$$

We therefore need an expression for the directional derivative $D_{e^{t\cdot}}(P; dP)$ of $P \to e^{tP}$. By definition of the matrix exponential, we have that

$$e^{t(P+dP)} = e^{tP} + \sum_{k=0}^{\infty} \frac{t^{k+1}}{(k+1)!} \sum_{j=0}^{k} P^{k-j}(dP)P^j + o(\|dP\|_{\mathcal{H}\to\mathcal{H}}). \tag{50}$$

The directional derivative is thus

$$D_{e^{t\cdot}}(P; dP) = \sum_{k=0}^{\infty} \frac{t^{k+1}}{(k+1)!} \sum_{j=0}^{k} P^{k-j}(dP)P^j. \tag{51}$$

By combining (49) and (51) we see that the directional derivative of f is

$$D_f(P; dP) = 2\left\langle e^{tP}\boldsymbol{q} - \boldsymbol{p}, \left(\sum_{k=0}^{\infty} \frac{t^{k+1}}{(k+1)!} \sum_{j=0}^{k} P^{k-j}(dP)P^j\right)\boldsymbol{q}\right\rangle_{\mathcal{H}} \tag{52}$$

$$= 2\sum_{k=0}^{\infty} \frac{t^{k+1}}{(k+1)!} \sum_{j=0}^{k} \langle e^{tP}\boldsymbol{q} - \boldsymbol{p}, (P^{k-j}(dP)P^j)\boldsymbol{q}\rangle_{\mathcal{H}}. \tag{53}$$

The final step is to derive G such that $D_f(P; dP) = \langle G, dP \rangle_F$. Note that $\langle \boldsymbol{q}, A\boldsymbol{p} \rangle_{\mathcal{H}} = \langle M\boldsymbol{q}\boldsymbol{p}^\intercal, A \rangle_F$ for arbitrary $\boldsymbol{p}, \boldsymbol{q} \in \mathbb{R}^N$ and $A \in \mathbb{R}^{N\times N}$. Thus, (53) becomes

$$D_f(P; dP) = 2\sum_{k=0}^{\infty} \frac{t^{k+1}}{(k+1)!} \sum_{j=0}^{k} \langle e^{tP}\boldsymbol{q} - \boldsymbol{p}, (P^{k-j}(dP)P^j)\boldsymbol{q}\rangle_{\mathcal{H}} \tag{54}$$

$$= 2\sum_{k=0}^{\infty} \frac{t^{k+1}}{(k+1)!} \sum_{j=0}^{k} \langle (P^{k-j})^\intercal M(e^{tP}\boldsymbol{q} - \boldsymbol{p})\boldsymbol{q}^\intercal (P^j)^\intercal, dP\rangle_F. \tag{55}$$

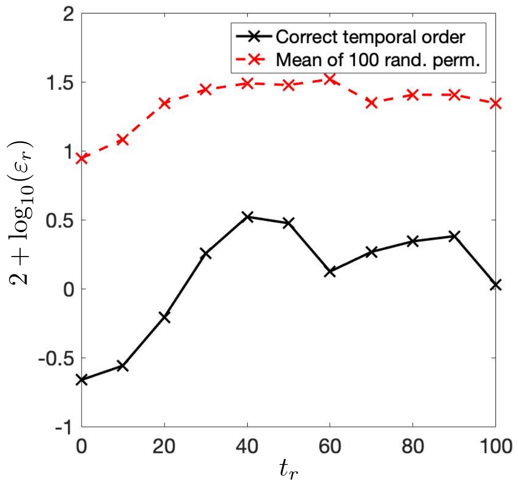

Fig S1. Dynamic distribution decomposition applied to data generated by stochastic dynamical system described by equation (1)–(2). Log transformed error plot plotting time point against $\log_{10}$ of the percentage error (of the DDD fit); the original data set (black solid line) has a mean error of 0.9%; the mean error over 100 permutations of the time labels (dashed red line) is 10%.

It therefore follows that the gradient of f at P is the matrix

$$G = 2\sum_{k=0}^{\infty}\frac{t^{k+1}}{(k+1)!}\sum_{j=0}^{k}(P^{k-j})^{\intercal}M(e^{tP}\boldsymbol{q}-\boldsymbol{p})\boldsymbol{q}^{\intercal}(P^{j})^{\intercal} \tag{56}$$

$$= 2\sum_{k=0}^{\infty}\frac{t^{k+1}}{(k+1)!}S_k\,. \tag{57}$$

The terms

$$S_k = \sum_{j=0}^{k}(P^{k-j})^{\intercal}M(e^{tP}\boldsymbol{q}-\boldsymbol{p})\boldsymbol{q}^{\intercal}(P^{j})^{\intercal}\,, \tag{58}$$

can be calculated using the recursion relation

$$S_k = P^{\intercal}S_{k-1} + S_{k-1}P^{\intercal} - P^{\intercal}S_{k-2}P^{\intercal}, \quad \text{where} \quad S_{-1} = \boldsymbol{0}\,,\, S_0 = M(e^{tP}\boldsymbol{q}-\boldsymbol{p})\boldsymbol{q}^{\intercal}\,. \tag{59}$$

Detecting Non-Autonomy

DDD can be used to detect non-autonomy. Repeating the SDE example from the Results section, we now change the observation times to $t = 0, 10, \ldots, 100$ and apply DDD. The resulting errors at each time point are plotted in Figure S1. We remind the reader of the earlier statement specifying that the increase error towards the end of the simulation is due to the trajectories interacting with the boundary conditions — boundary conditions that are not incorporated into the choice in basis functions.

We then randomly reorder the time labels such that the data now appears out of sequence, then apply DDD. Performing this 100 times and taking the mean error at each time point, we find that this new error is approximately constant over time. When compared to applying DDD to the data in its correct temporal sequence, the reordered data has approximately an order of magnitude larger error.

Therefore, one can conclude that DDD fitting error reflects non-autonomy, and higher fitting error indicates when this assumption is breaking.
