# Supplementary material for: Dynamic distribution decomposition for single-cell snapshot time series identifies subpopulations and trajectories during iPSC reprogramming
Source: PLoS Comput Biol. 2020 Jan 10;16(1):e1007491. doi: 10.1371/journal.pcbi.1007491 (PMC6953770; doi:10.1371/journal.pcbi.1007491)
Supplement: S2 Appendix — A document containing two additional figures: protein expression of Nanog-Neo cell line data taken from Zunder et. al.; and plots containing visualisations of the coefficient vectors cr and mass matrix M for both the the stochastic dynamical system and Nanog-Neo cell line data. (PDF) [file pcbi.1007491.s002.pdf]

# S2 Appendix: Additional Figures

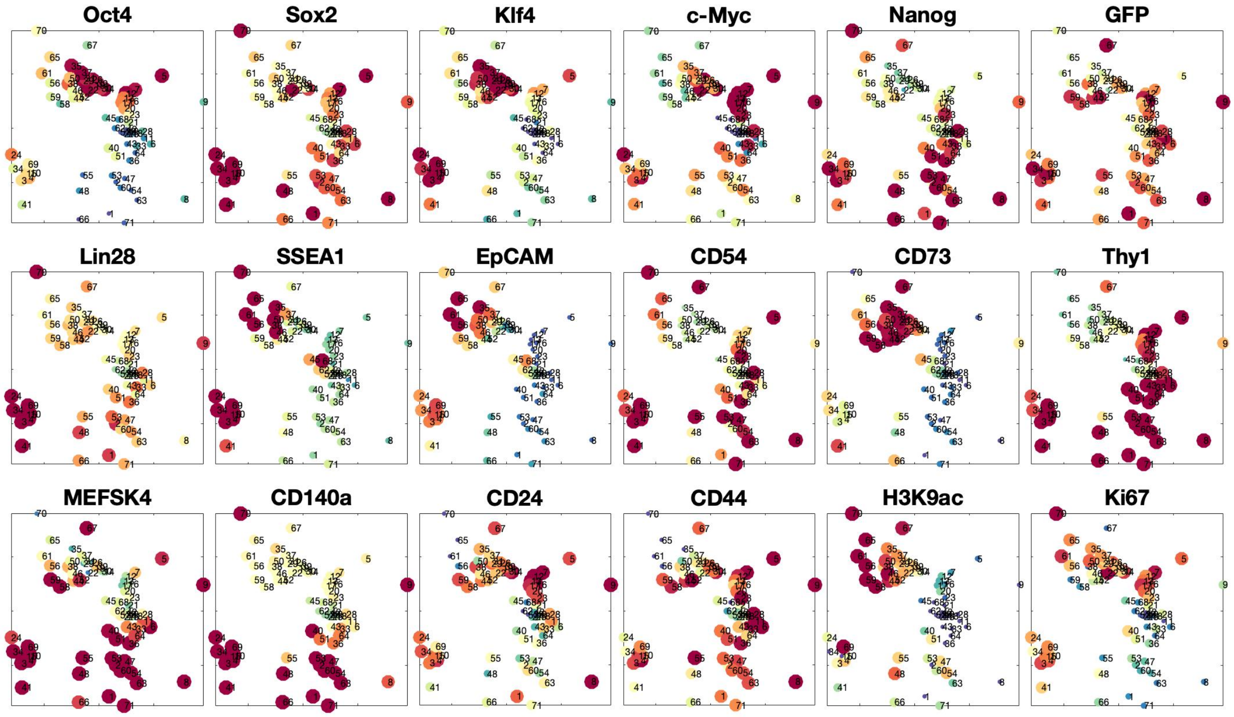


**Fig S2.** Protein expression of Nanog-Neo cell line data taken from Zunder *et. al.* [28]. Locations are plotted using positions of nodes in Figure 6. After the raw data is transformed via $f(x) = \text{arcsinh}(x/5)$ and $z$-scored, we limit the colour scale to the middle 50% of the data (lower quartile in dark blue, upper quartile in dark red).

Figure S2 shows the expression levels of the 18 protein markers at each of the locations of the basis functions after fitting to the Zunder *et. al.* [28] data set. Figures S3 and S4 shows visualisations of the entries of the coefficient vectors $\boldsymbol{c}_r$ for each time point and the inferred initial state $\boldsymbol{c}_*$; and the mass matrix $M$ associated to the basis functions $\{\psi_j\}_{j=1}^N$. For Figures S3(a) and S4(a), bold black lines around entries of $\{\boldsymbol{c}_r\}_{r=1}^R$ show basis points which were added at each time point.

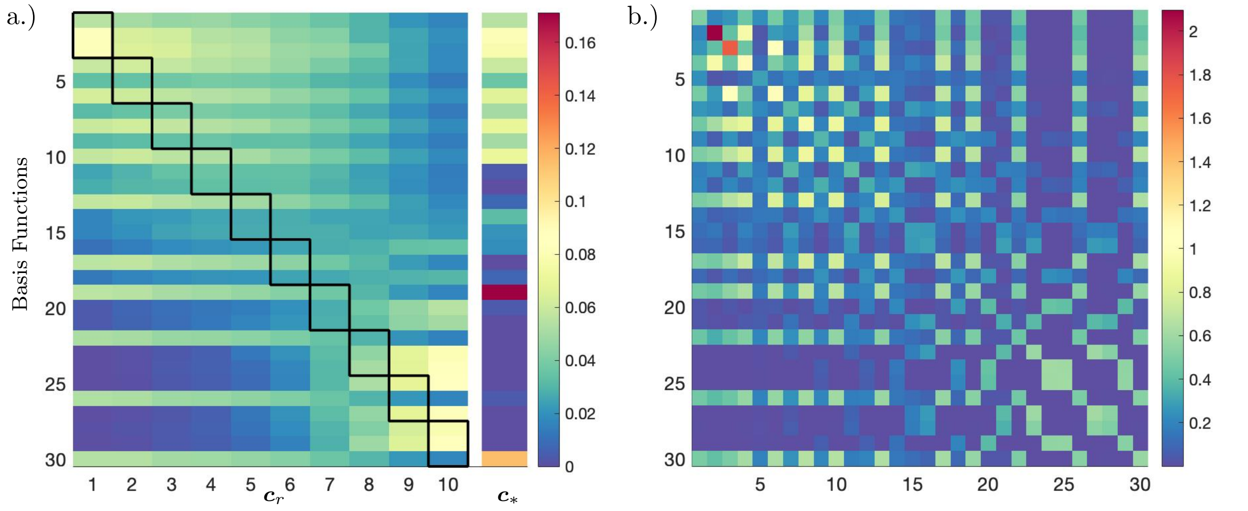


**Fig S3.** Dynamic distribution decomposition applied to data generated by stochastic dynamical system described by equation (1)–(2). For Visualisation of the entries of (a.) the coefficient vectors $\boldsymbol{c}_r$ for $r = 1, \ldots, R$ and $\boldsymbol{c}_*$; and (b.) the mass matrix $M$.

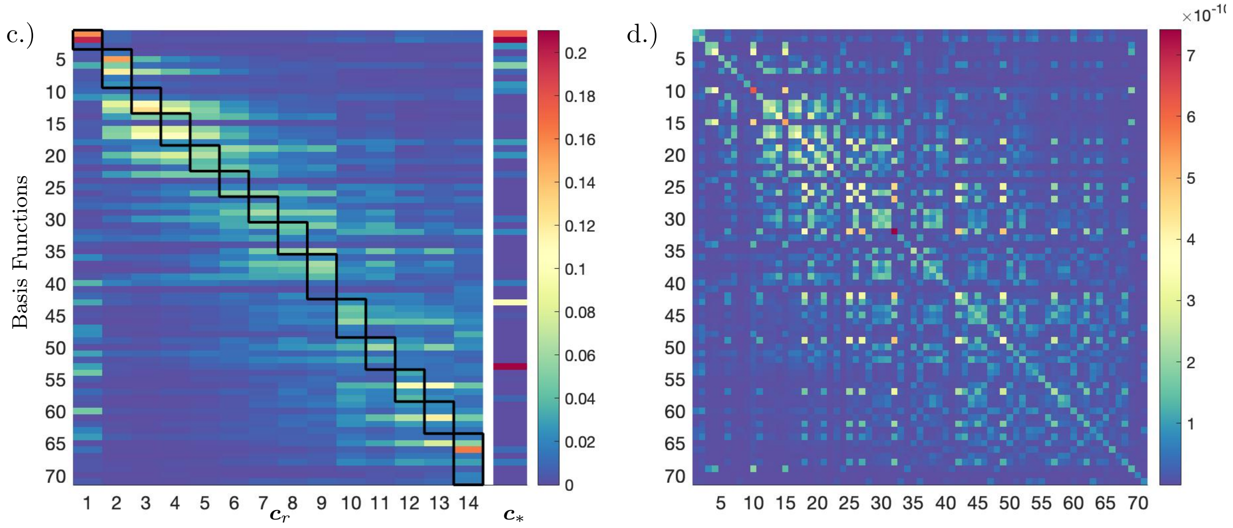


**Fig S4.** Dynamic distribution decomposition applied to Nanog-Neo cell line data taken from Zunder *et. al.* [28]. Visualisation of the entries of (a.) the coefficient vectors $\boldsymbol{c}_r$ for $r = 1, \ldots, R$ and $\boldsymbol{c}_*$; and (b.) the mass matrix $M$.
