# Supplementary material for: Dynamic distribution decomposition for single-cell snapshot time series identifies subpopulations and trajectories during iPSC reprogramming
Source: PLoS Comput Biol. 2020 Jan 10;16(1):e1007491. doi: 10.1371/journal.pcbi.1007491 (PMC6953770; doi:10.1371/journal.pcbi.1007491)
Supplement: S3 Appendix — A document detailing a brief comparison between our method and 3 other published methods: Wishbone [42], Monocle2 [43], and Waddington-OT [6]. (PDF) [file pcbi.1007491.s003.pdf]

S3 Appendix: Comparison with other Established Methods

We now perform a brief comparison between our method and 3 other published methods: WISHBONE [42], MONOCLE2 [43], and WADDINGTON-OT [6]. We note that many of these algorithms were created primarily for the use of single cell RNA-Seq data, i.e., $\mathcal{O}(10^3)$ collected cells with $\mathcal{O}(10^3)$ numbers of features. However mass cytometry data can collect $\mathcal{O}(10^5)$ cells whilst only recording $\mathcal{O}(10^2)$ numbers of features. With regards to algorithms MONOCLE2 and WADDINGTON-OT, RAM and storage requirements necessitated subsampling of the full dataset: we selected 1000 samples from each of the 14 time points totalling 14000 samples. We note that WISHBONE and our DDD approach do not have this limitation. In fact for our method once basis functions are chosen and $\{\boldsymbol{c}_r\}_{r=1}^{R}$ are calculated, the method is independent of the number of data points.

WISHBONE and MONOCLE2

Both WISHBONE [42] and MONOCLE2 [43] are pseudotime ordering methods: provided with a gene-by-cell matrix these algorithms will search for structures in the data and infer an order from some starting point. In our case, the starting point was chosen to be cells with an Oct-4$^+$, KLF4$^+$expression profile. For us to apply these methods, the time label for each cell was omitted.

To assess the quality of the pseudotime ordering we plot each cell's harvest time label against the pseudotime value; for both WISHBONE and MONOCLE2 this lead to uncorrelated plots, see Figures S5 and S6. Were it the case that these pseudotime ordering methods worked well on time series data, we would see a monotonic relationship between both variables. Clearly this is not the case, and therefore we question the benefit of using such a method on time series data. The same conclusion was reached in Schiebinger *et. al.* [6].

WISHBONE was not able to elucidate on any structure within the data and therefore we focused on MONOCLE2. After application of MONOCLE2, three branching points were identified, see Figure S6. However, it appears that all three are biologically unrealistic as they have CD73$^+$, CD140$^-$expression profiles that are inconsistent with Zunder *et. al.* [28].

WADDINGTON-OT

The method developed by Schiebinger *et. al.* [6], known as WADDINGTON-OT, appears to be most similar to DDD in that it is specifically designed for time series data. WADDINGTON-OT uses Optimal Transport to map between pairs of subsequent time series measurements by minimising the "Earthmover's distance", i.e., how far each unit of probability mass needs to be displaced. However, instead of encoding data points

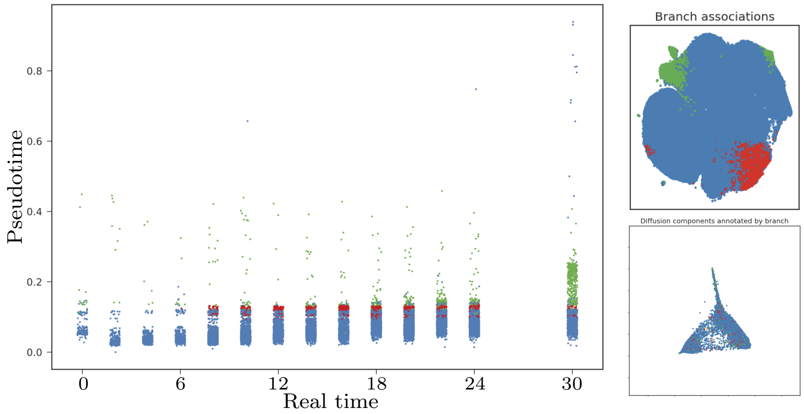

Fig S5. WISHBONE [42] applied to Nanog-Neo cell line data taken from Zunder *et. al.* [28].

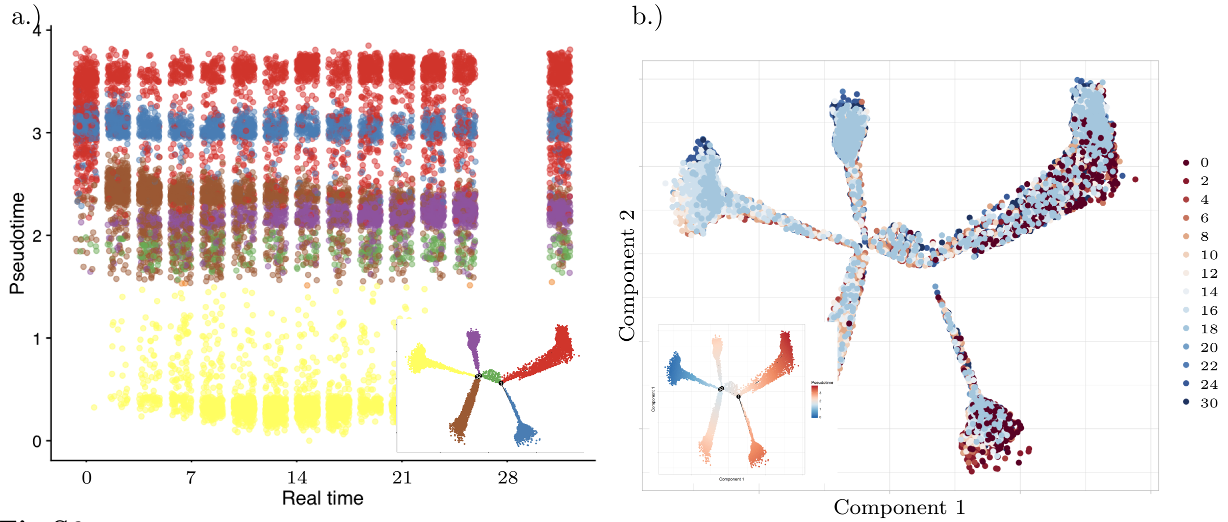

Fig S6. MONOCLE2 [43] applied to Nanog-Neo cell line data taken from Zunder *et. al.* [28].

into distributions, they map individual data points together from one time point to the next. Combining the $R-1$ pairwise maps together leads to the creation of a weighted directed graph estimating how the data points at one time point map to data points at the preceding and following times. Downstream analysis can then be carried out; their suggestions included: clustering cells together and looking at maps between these clusters; and fitting models using these maps to infer gene regulatory networks.

While their work suggested how to calculate "decendants" and "ancestors" for a particular cell in question, one point left unaddressed was how to calculate branching points from their maps. This is the point of comparison we would like to use between methodologies. To overcome this difficulty, we calculated the betweenness node centrality measure indicating nodes which frequently lay on paths between other pairs of nodes. To test this method worked, we applied it to the simulated data to find a single clear branching point at location $(0.66, 0.37)^\intercal$ which is a reasonable estimate and consistent with the DDD estimate of $(0.65, 0.64)^\intercal$ as annotated in Figure 4.

We then used this same approach on the data generated by Zunder *et. al.* [28], plotted using the force directed layout from WADDINGTON-OT [6] in Figure S7. From this procedure 3 data points had exceedingly high betweenness centrality, see Figure S7(b): the first and second data points were associated to times $t = 4$ days and $t = 6$ days and have similar expression profiles to basis function 16 as found from our earlier analysis; the final data point with high betweenness centrality was found at $t = 14$ days but did not resemble the expression profile as described by basis function 29 and previously within the original paper by Zunder *et. al.* [28].

We hypothesise that our method will perform better than WADDINGTON-OT for datasets where: i.) there is a large number of measurements (WADDINGTON-OT can generate large files); ii.) there are erratic/large gaps of time between measurements (WADDINGTON-OT assumes small gaps between measurements); and/or iii.) the system is believed to be autonomous.

a.) b.)

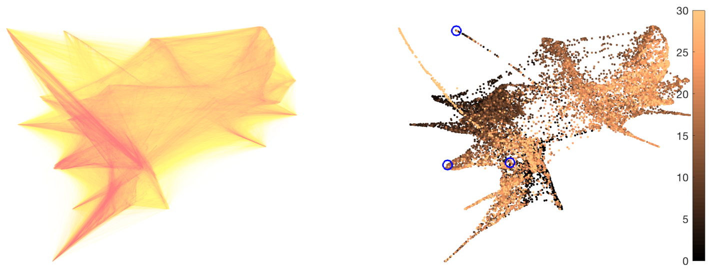

Fig S7. Waddington-OT [6] applied to Nanog-Neo cell line data taken from Zunder *et. al.* [28]. Using the force directed graph layout, we plot: (a.) edges between nodes; (b.) nodes only using time labels.
