# Supplementary material for: Dynamic distribution decomposition for single-cell snapshot time series identifies subpopulations and trajectories during iPSC reprogramming
Source: PLoS Comput Biol. 2020 Jan 10;16(1):e1007491. doi: 10.1371/journal.pcbi.1007491 (PMC6953770; doi:10.1371/journal.pcbi.1007491)
Supplement: S4 Appendix — A document detailing DDD applied to an additional control dataset with known underlying cellular behaviour, see Lun et. al. [32]. (PDF) [file pcbi.1007491.s004.pdf]

S4 Appendix: Reconstruction of EGFR Signalling from Mass Cytometry Time Series Data

We apply DDD to an additional dataset where we know the underlying cellular behaviour. We apply DDD to one of the control experiments originally analysed in Lun *et. al.* [32]. In their work, under the influence of perturbations (via transfected plasmids) the EGFR signalling network in HEK293T cells was examined by mass cytometry. Five measurements were then taken at 0, 5, 15, 30, and 60 minutes. For such a control experiment, cells were transfected by an empty plasmid and as such: the distribution in the state space does not change, except for a change in ERK expression.

We choose basis functions as described before. To reduce dimensionality we consider the as input the principle components accounting for 90% of the variance within the data. We apply DDD to recover a Markov transition matrix (17% mean fitting error), see Figure S8(a), and apply the Lasso regularisation, see Figure S8(b). Examining eigenfunctions (not plotted) to find key states at basis functions 21 and 33, the Lasso regularisation can be used to infer a straight progression between basis functions 6→16→21→33. When viewing the corresponding expression ERK for each basis function in Figure S8(c), we see that we move from a state of low ERK expression to one of high ERK expression before returning to the original low expression state. The experimental time series measurements of mean ERK approximately agree with this.

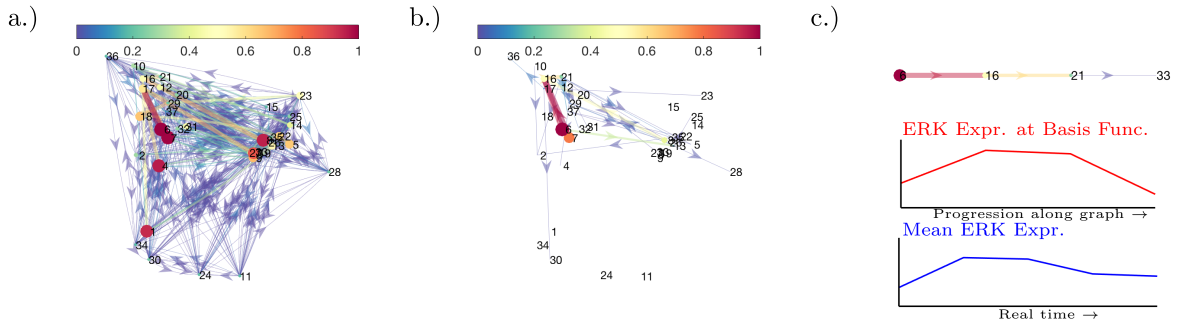

Fig S8. Dynamic distribution decomposition applied to the control experiment in Lun *et. al.* [32]. (a.) Markov transition matrix P plotted without Lasso regularisation; (b.) Markov transition matrix P plotted with Lasso regularisation, $\beta = 1/[5000 \times \text{mean}(M)]$, see Methods section; and (c.) Simplified graph of transitions between eigenfunction extreme, basis function location of ERK and measured ERK time series.
